# Safety of higher dosages of *Viscum album *L. in animals and humans - systematic review of immune changes and safety parameters

**DOI:** 10.1186/1472-6882-11-72

**Published:** 2011-08-28

**Authors:** Gunver S Kienle, Renate Grugel, Helmut Kiene

**Affiliations:** 1 Institute for Applied Epistemology and Medical Methodology at the University of Witten/Herdecke, Zechenweg 6, 79111 Freiburg, Germany

## Abstract

**Background:**

*Viscum album *L extracts (VAE, mistletoe) and isolated mistletoe lectins (ML) have immunostimulating properties and a strong dose-dependent cytotoxic activity. They are frequently used in complementary cancer treatment, mainly to improve quality of life, but partly also to influence tumour growth, especially by injecting VAE locally and in high dosage. The question is raised whether these higher dosages can induce any harm or immunosuppressive effects.

**Methods:**

Systematic review of all experiments and clinical studies investigating higher dosages of VAE in animals and humans (*Viscum album *> 1 mg in humans corresponding to > 0.02 mg/kg in animals or ML > 1 ng/kg) and assessing immune parameters or infections or adverse drug reactions.

**Results:**

69 clinical studies and 48 animal experiments reported application of higher doses of VAE or ML and had assessed immune changes and/or harm. In these studies, *Viscum album *was applied in dosages up to 1500 mg in humans and 1400 mg/kg in animals, ML was applied up to 6.4 μg/kg in humans and in animals up to 14 μg/kg subcutaneously, 50 μg/kg nasally and 500 μg/kg orally. A variety of immune parameters showed fluctuating or rising outcomes, but no immunosuppressive effect. Side effects consisted mainly of dose-dependent flu-like symptoms (FLS), fever, local reactions at the injection site and various mild unspecific effects. Occasionally, allergic reactions were reported. After application of high doses of recombinant ML, reversible hepatotoxicity was observed in some cases.

**Conclusions:**

Application of higher dosages of VAE or ML is not accompanied by immunosuppression; altogether VAE seems to exhibit low risk but should be monitored by clinicians when applied in high dosages.

## Background

Complementary cancer treatment is utilised by 15-73% of all cancer patients in Europe, in addition to well established oncological treatments [1]. Most of these complementary treatments are herbal remedies and among these, *Viscum album *L extracts (VAE, European mistletoe, a hemiparasitic shrub, not to be confused with the *Phoradendron *species or "American mistletoe") are frequently used [1]. Physicians in Germany consider VAE to have a relevant therapeutic benefit [2].

VAE contains a variety of biologically active compounds. Of these, mistletoe lectins (ML I, II and III) have been most thoroughly investigated. ML consist of two polypeptide chains: a carbohydrate-binding B-chain that can bind to cell surface receptors and thus enable the protein to enter the cell [3-5]; and the catalytic A-chain which can subsequently inhibit protein synthesis, due to its ribosome-inactivating properties, by removing an adenine residue from the 28S RNA of the 60S subunit of the ribosome [3]. Other pharmacologically relevant VAE compounds are viscotoxins and other low molecular proteins, VisalbCBA (*Viscum album *chitin-binding agglutinin) [6], oligo- and polysaccharides [7,8], flavonoids [9], vesicles [10], triterpene acids [11], and others [12,13].

Whole VAE as well as several of the compounds are cytotoxic and the ML in particular have strong apoptosis-inducing effects [14-16] and also stimulate the immune system (*in vivo *and *in vitro *activation of monocytes/macrophages, granulocytes, natural killer (NK) cells, T-cells, dendritic cells, induction of a variety of cytokines) [12,13].

For clinical application, VAE are made from mistletoe grown on different host trees (table 1). Depending on the host tree, the harvesting time and the extraction procedure, VAE vary in regard to their active compounds and biological properties. Different commercial VAE preparations are available, and a recombinant ML (rML) drug is currently being developed and tested in animals [17-19] and in clinical trials [20-22].

**Table 1:** Host trees of *Viscum album*, used in medical preparations

Fir	*Abies*	(A)
Maple	*Acer*	(Ac)
Almond	*Amygdalus*	(Am)
Birch	*Betula*	(B)
Hawthorn	*Crataegus*	(C)
Ash	*Fraxinus*	(F)
Apple	*Malus*	(M)
Pine	*Pinus*	(P)
Poplar	*Populus*	(Po)
Oak	*Quercus*	(Qu)
Willow	*Salix*	(S)
Lime	*Tilia*	(T)
Elm	*Ulmus*	(U)

Effectiveness and efficacy of VAE have been assessed in various systematic reviews [23-30]. Safety aspects, besides being secondary outcomes in these reviews, were assessed systematically in five specific reviews: two on adverse reactions [31,32], one on toxicology [33], and one each in a health technology assessment (HTA) report [34] and in a comprehensive review on VAE research [35].

In cancer therapy, VAE are usually applied at a rather low dosage, adjusted individually according to local reactions at the injection site (LR) and individual tolerability. Increasingly, however, VAE are also applied in high dosages, either intratumourally, systemically (subcutaneously), or as an intravenous infusion to achieve tumour remission or to substantially improve quality of life [36-40].

As VAE not only have immunostimulatory but also cytotoxic properties - some of its components, like the ML, are highly cytotoxic, comparable to conventional cytotoxic agents [41-45] - the question is raised whether VAE in high dosage can lead to severe side effects and, specifically, whether they suppress immune functions, which is a well-known detrimental effect of other cytotoxic anticancer drugs. This question was raised in a casuistic report achieving tumour remission with high dosage VAE infusion (up to 700 mg Helixor) [46] and in an animal experiment applying 2.5 and 5 ng ML I/kg body weight (BW) in mice [47].

Tolerability of high dosage VAE has not yet been assessed systematically in any of the above mentioned reviews, especially not the question of immunosuppression. Still, in a variety of animal experiments and clinical studies, high doses of VAE or ML have been applied and haematological, immunological and general safety outcomes assessed. We decided to systematically review these clinical studies and animal experiments to assess the following questions: Do VAE or ML have immunosuppressive effects when they are applied at higher therapeutic dosages? Which other side effects have been observed in studies or experiments when VAE or ML are applied at higher dosages? Are these dose-dependent? - Purely toxicologic tests were not reviewed, as they have already been reviewed elsewhere [33]. Also, these tests employ ultrahigh and lethal dosages to provoke toxic effects, which are not used therapeutically; their review would require a different methodology and would have to cover other material as well (e.g. in vitro tests).

As VAE-induced immunostimulation is observed in low dosages of 1 mg Viscum album and 1 ng ML/kg BW, which is generally considered to be an immunostimulating dosage, we set these dosages as a threshold for "higher dosage", although this is somewhat arbitrary.

## Methods

### Design

Systematic review of clinical studies and animal experiments. Methodology and presentation adhere to PRISMA guidelines [48] and to recommendations for conducting systematic reviews of adverse effects [49].

### Identification of secondary immunosuppression

Immunosuppression is one category of immunotoxic effects - encompassing also hypersensitivity, autoimmunity and adverse immunostimulation [50,51]. Immunosuppression refers to a profound impairment of the immune response leading to an increased susceptibility to infectious micro-organisms, partly with a specific viral or bacterial spectrum depending on the type of immune defect, and to more frequent neoplasias, in particular lymphomas [50,52]. Secondary immune defects - far more common than primary immunodeficiencies, resulting from inborn genetic defects - are caused by a variety of factors like drugs (e.g. anticancer cytotoxic agents, corticesteroids, some antibiotics), radiation, and various illnesses (HIV-infection, malignant or metabolic diseases, protein-losing syndrome, severe malnutrition, polytrauma, excessive physical stress) [50,53].

To assess immunotoxic effects of drugs, a tiered approach is generally recommended. The first step consists of observations from clinical or standard non-clinical toxicology studies, leading to the suspicion of immunosuppression: i.e. increased infections, increased incidence of neoplasias, abnormal results of complete and differential blood count (CBC and DBC) or serum immunoglobulins. An optional or second step would assess delayed type hypersensitivity (DTH), and, in case of standard toxicological studies, weight and histology of immune organs. If these are clearly abnormal, further specific immunological tests are recommended [54].

A detailed algorithmic screening was proposed as follows [55]. Step 1: Anamnesis (increased susceptibility to infections, at least 3 periods of infections per year, each of more than four weeks duration [56]), CBC, DBC, and quantitative determination of immunoglobulins. If findings are abnormal, step 2: T-cells, thymus size, DTH, T-cell phenotyping, lymphocyte transformation test. If findings are abnormal, step 3: B-cells, IgG subclasses in case of IgA deficiency, isohaemagglutinin, immunisation. Subsequently step 4: complement, phagocytic activity. And, when indicated, step 5: molecular biological testing. If no clinical symptoms or clear pathological alteration of these immune parameters are present, further elaborate analyses of immune functions are of questionable use and may be misleading [55-58].

Fluctuating immune parameters are normal to a large extent [54,59]. Furthermore, transient, short-term changes of immune parameters connected to acute stress, including application of very high doses of pharmaceuticals, are well-known; they are not correlated to subsequent immunosuppression but to immunostimulation [60,61]. (See also discussion section.)

According to the above mentioned recommendations we screened clinical and animal studies with regard to whether clinical signs or a clear and consistent pathological alteration of classical immune parameters were observed during higher doses of VAE or ML.

### Identification of adverse drug reactions (ADRs)

An adverse drug reaction is an untoward event presenting during drug treatment at doses normally used in humans and for which a causal relationship between the drug and the event is at least a reasonable possibility, according to the judgement of the reporting or reviewing health care professional. For this review we screened all trials, studies, case-series and animal experiments applying higher dosages for reports of ADRs (e.g. "side effects", "adverse events", "adverse effects", "adverse reactions", "ADR", "toxicity", "safety", "tolerability", "laboratory changes", "abnormalities", "systemic symptoms"). Causal relationship was not additionally assessed if this was already done by the reporting health care professional.

### Search strategy

We used a systematic process to search eight databases for clinical studies and animal experiments - from inception of these databases to February 2011. For details (databases, search terms, search strategies, results) see Additional file 1: *Literature Search*. The reference list from each potentially eligible study, relevant review article and textbook was checked, and experts in the field and manufacturers of mistletoe preparations were contacted for additional reports.

**Figure 1:**
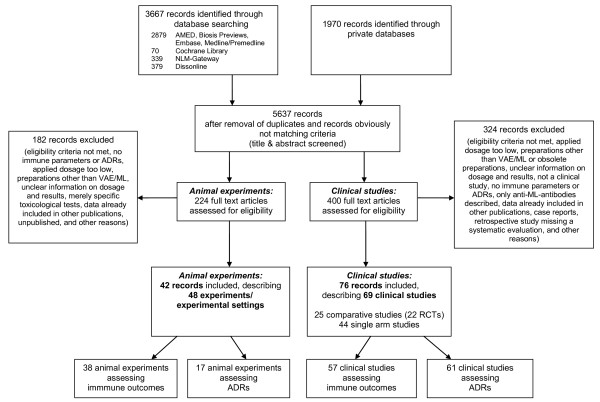
**Literature search and study selection**.

As no single index or subheading search term reliably identifies data on adverse effects or immunosuppression [49], all trials, clinical studies, case series, and animal experiments were individually checked for inclusion criteria. They were screened by two independent researchers (GSK, RG) for identification of eligibility criteria, especially applied dosage and whether ADRs were reported or immune parameters or episodes of infections were assessed. The study selection process is presented in Figure 1.

### Eligibility criteria

The following selection criteria were used for inclusion of studies in the analysis: (I) clinical study (prospective randomised controlled trial, RCT, or non-randomised controlled study, or prospective single-arm cohort study, or phase II trial, or case series) or animal experiment; (II) study population with any disease or without diseases; (III) intervention group treated with VAE preparation dosed at > 1 mg Viscum album, corresponding to 0.02 mg/kg BW in animals, or > 1 ng ML content/kg BW or with isolated or recombinant ML dosed at > 1 ng/kg BW; (IV) outcome measure: immune parameter (i.e. white blood cells, lymphocytes, granulocytes, immunoglobulins, complement factors, cytokines, cellular or humoral immune response, weight and histology of immune organs; studies only presenting anti-ML antibodies were not included) or ADRs; (V) completion of study or interim report; (VI) published or unpublished. There were no restrictions on language. For animal experiments, unpublished material was not included. Purely toxicologic tests were not included (for review see [33]).

### Validity assessment and data abstraction

As we are conducting a widely scoped review [49] and also because we are searching for rare effects [49] and for effects present under higher dosage and alternative application forms which are investigated mainly in single-arm studies and animal experiments, we included different kinds of clinical studies, irrespective of their methodological design and quality, and also animal experiments. Study design, detecting methods and reporting quality play a key role concerning the risk of bias when side effects are investigated in clinical or animal studies; beyond this, however, there is no validated, reliable quality assessment tool for the analysis of adverse effects [49]. We therefore critically discussed and appraised all studies individually. They were classified according to design, especially pertaining to whether they compared the outcome to a control group or to the status before intervention; whether the allocation of the intervention had been randomised; whether the treatment application had been blinded; and whether participants were healthy or not. Methods used to detect immune changes or adverse effects were assessed as well as the duration of follow-up, co-interventions potentially influencing the outcomes, reporting of results as well as general study characteristics: whether they were prone to bias, inflating or deflating potential side effects, drop outs and general publication quality. Corresponding results were presented when they had an influence on the outcome of interest (for other outcomes, see [23-26]).

Data on participants, intervention, co-intervention, detecting methods, and outcomes referring to safety and the immune system (except anti-ML-antibodies) were abstracted in evidence tables by one researcher and checked by a second (GSK, RG). ML content of VAE was obtained from the respective publication, or, if not available, it was either left out, or calculated from other contemporary publications on the same preparation, or calculated on the basis of information provided by the corresponding manufacturer. This latter information is based on average values from different batches and includes a degree of fluctuation. One has to be aware that, firstly, the dosages of different preparations cannot be compared, due to different extraction procedures; that, secondly, the ML content of different preparations cannot be compared as they are measured with different assays (e.g. ELLA, ELISA [62]) and have different proportions of ML I and ML II/III; and that, lastly, lectins are only part of the oncologically active ingredients of VAE. The indication of the lectin content shall only serve as an approximate orientation, and does not claim precision nor sole clinical relevance. This is particularly the case for quantification of ML dosages in older studies (before ~ 1990) which often used different, non-standardised methods and therefore do not allow comparison.

Besides the included studies, also all excluded studies were screened for major adverse events. As the studies have a high heterogeneity in regard to design, comparison group, modalities of therapy, assessment of immune changes and ADRs, analysis, and reporting, we considered that a quantification of results, e.g. relative risk, would be highly imprecise and misleading. Therefore the information was summarised in a qualitative and descriptive manner in tables and in the text.

## Results

### Clinical studies - selection and characteristics

Regarding literature search and study selection see Figure 1 and Additional file 1. Altogether, 69 clinical studies matched inclusion criteria. Their characteristics are summarised in table 2 and their details are presented in Additional file 2. The methodological quality of the studies varied substantially (see table 2 and Additional file 2, for further quality aspects of the clinical studies, see [12,24-26]). 10 of the studies were conducted following modern standards of *good clinical practice*. The immunological and safety outcomes of the studies were presented only partly numerically and in detail; often they were summarised, just mentioned in the conclusion section or the abstract or not reported. Missing patients were often not reported for immunological or toxicity outcome.

**Table 2:** Characteristics of the clinical studies (n = 69)

	Number of studies
**Types of study**	
Controlled studies, comparing outcome to control group (2912 patients included)	25
Randomized	22
Double blind	6
Single blind	1
Single-arm studies, comparing outcome pre-post (1347 patients included)	44

**Diagnoses**	
Healthy participants	10
Cancer	48
Others: Hepatitis C, immunosuppression, HIV infection, osteoarthritis, anal condyloma (4 × mixed: HIV & healthy; 1 × mixed: HIV & healthy & cancer)	11

**Treatment**	
Whole extract	66
Recombinant ML	3

**Application route**	
Subcutan, intracutan	50
Intravenous *	10
Intrapleural, intraperitoneal, intravesical *	7
Intratumoural *	2

**Application frequency**	
Applied just once	7
Applied more than once in constant dosage (up to 3 years)	12
Applied more than once in escalating dosage (up to 6 years)	50

**Maximum dose per application**	
≤ 20 mg VAE	36
> 20 - 100 mg VAE	15
> 100 mg VAE (maximum dose: 1500 mg; maximum ML content: 45000 systemically, 250000 ng intravesically)	15
> 100000 ng rML (maximum dose: 448000 ng)	2
< 100000 ng rML	1

**Observation time < 1 month**	**14**

**Treatment of control group (n = 25)**	
No additional treatment	14
Placebo	6
Active (multivitamins, Lentinan, Etoposide, BCG, non-stimulating skin control test/immignost)	5

**Immune outcomes investigated**	**57**
Clinical infections	4
Peripheral blood: CBC, DBC, lymphocytes & subsets, mitogen-induced proliferation, cytokine release, NK-cells & activity, ADCC, phagocytosis of granulocytes, cytokines; immunoglobulins, CRP, haptoglobin, others	55
Immune parameters in tumour tissue, pleural effusion, saliva, urine	6

**Safety outcomes investigated**	**61**
Safety as primary objective of the study	6
Systematic and regular assessment of clinical and laboratory parameter (electrolytes, urea, AST, ALT, γ-GT, AP, bilirubin, creatinine, creatine kinase, LDH, protein, albumin, glucose, cholesterol, triglycerides, α-amylase)	29
Recorded according to NCI CTC, WHO toxicity criteria, Likert scale, Lilly tables	17
Other modalities of recording	16
No details on recording	16

**Time schedule of safety assessment**	
Daily (e.g. diary)	6
Weekly, biweekly	12
Monthly, every 3 weeks	13
Quarterly	4
Once	2
„Regular"	7
No details or no systematic plan	25

* Partly concomitant sc application

**Abbreviations**: ADCC: antibody-dependent-cell-mediated cytotoxicity, ALT: Alanine transaminase, AP: alkaline phosphatase, AST: aspartate transaminase, CBC complete blood count, CRP: C-reactive protein, DBC: differential blood count, γ-GT: γ-glutamyltransferase, LDH: lactate dehydrogenase, NCI CTC: National Cancer Institute - Common Toxicity Criteria

### Clinical studies - immunological results (see Additional file 2)

Most studies reported an increase of the immune parameters during or after VAE/ML application, some reported no significant difference and some a decrease. In detail:

*Infections: *A dose-dependent (maximum 5 mg) increase of episodes was observed in HIV positive patients [63,64], however without being accompanied by a deterioration of immune parameters and therefore without any signs of immunosuppression. All other studies showed a decreased incidence and duration of infections - respiratory infections in immunosuppressed children (due to the Chernobyl nuclear accident) [65], or common colds in healthy subjects [66,67].

*Haematology (leukocytes): *One study applying escalating doses of recombinant ML - up to 6400 ng/kg - observed a slight and transient leukocytopenia (common toxicity criteria, NCI CTC, grade 1) during the first cycle in four of the 41 patients with advanced, refractory progressive cancer [20]. In subsequent treatment cycles as well as in a subsequent study, the same recombinant ML was applied in the same dosage, but no leukocytopenia and no other haematological toxicity were observed [20,21]. In two other studies (without a control group), one described a slight, transient, non-significant decrease of leukocytes after month 6 in cancer patients (to an average of 5281 cells/μl, standard deviation: 1145) [68], the other described a decrease of leukocytes (to a minimum of 5500 cells/μl) in patients with a prior leukocytosis (and, vice versa, an increase of leukocytes after prior leukopenia) [69]. All other studies showed either an increase, or no difference or an improved recovery from chemo- or radiotherapeutic toxicity. So, no indication of haematologic toxicity or granulocytopenia was observed.

*Lymphocytes: *A reduction was described in some of the studies: In one study lymphocytes decreased (to a minimum of 3500 cells/μl, standard error: 340) in patients with an initial leukocytosis, while patients with initially low values showed rising lymphocytes [70]. In other studies either a reduction of lymphocytes within normal range or an initial, transient reduction after starting VAE treatment was mentioned. In one comparative study, reduced lymphocytes were observed in patients applying VAE concomitantly to chemotherapy, especially when the patients had additional glucocorticoids; here, groups were not easily comparable; they particularly differed with regard to co-therapy, and no adjustment was made for patients' imbalances [71]. Two further studies found a reduction of CD8 cells (interpreted as suppressor cells), while other lymphocyte subsets increased [72-74]. Finally, one study found a slight decrease of the proportion of CD3+T-cells expressing CD25 after mitogen (PHA) stimulation ("activated" T-cells) during 6 months of VAE treatment in cancer patients; the patients were treated either by a physician preferring a *slow *or by another physician preferring a *swift *VAE dosage escalation, and the respective decrease was more pronounced during the swift dose escalation ("swift" group: from 80.8 to 74.4%; "slow" group: from 74.6 to 69.9%), but did not depend on the dosage actually applied. These decreases were, however, still within the range of healthy controls (72.9%). Besides, the proportion of HLA-DR+CD3+ T-cells decreased in the "swift" group but not in the "slow" escalation group [75].

*Immunoglobulins: *An increase was observed in two studies [65,74]; specific anti-ML-antibody production was assessed in 10 of the reviewed studies (data not shown).

*Cytokines: *The studies showed highly variable results, with mostly increase, but also decrease of the corresponding cytokine endpoint, without any indication for immunosuppression.

Remaining parameters - *functional capacity of granulocytes, NK-cell number and activity, peritumoural and tumour infiltration, VAE- or mitogen-induced proliferation or cytokine release of peripheral blood mononuclear cell*s, *complement factors, acute phase proteins, ADCC: *The studies found either an increase or no significant change, or an initial transient decrease (NK and ADCC activity) in one study [76,77].

Altogether in these 57 studies no indication for an immunosuppression through VAE or ML was found, not even when high doses of VAE or ML were applied. Especially the studies that compared outcomes to a control group found no immunosuppressive effects.

### Clinical studies - ADRs (see Additional file 2)

Of the 58 studies mentioning safety assessment during VAE application, two did not report any results; 32 studies reported to have observed no ADRs, to have good tolerability or mainly LR; 20 studies reported to have observed well-known or unspecific and usually mild side effects or events with unclear relation to VAE application, like rise in temperature, fever, FLS, mild infections (see above), headache, lassitude, fatigue, dry eyes, flatulence/loose stool, nausea, moderate pain after intraperitoneal or intrapleural instillation, cutaneous rash, anxiety during sleep or slight changes of laboratory parameters within the normal range (see below). Of the remaining 4 studies, besides reporting good tolerability, one also reported grade 3 cellulitis, two studies an angioedema/urticaria occurring in two patients [78,79] - one of which occured only under higher dosage, and was no longer observed when dosage was reduced [79] - and one study also short-term ADRs in three patients (after intraperitoneal application of high doses of VAE) which were not specified [80]. Of the studies referring to laboratory parameters, two described a slight increase of urea and creatinine, and a slight decrease of albumin, haemoglobin and erythrocytes (HIV positive patients) - all within normal ranges - during several months of VAE application [63,64]. All other studies reported no deviations and particularly no organ toxicity.

Three phase I trials investigated the dose-limiting toxicity of an experimental compound, a recombinant ML (rML) preparation in patients with advanced cancer (with no control group to distinguish from disease symptoms or else). rML was applied as a 1 h or a 24 h central intravenous infusion in two studies, with dose levels ranging from 10 to 6400 ng/kg and from 4000 to 6000 ng/kg [20,21], and as an subcutaneous injection up to 10 ng/kg in the third trial [22]. rML was reported as being very well tolerated in all three studies. Most frequently observed were LR, fatigue, fever, nausea, vomiting, and high urinary frequency, usually grade 1-2. At 6000 and 6400 ng/kg, dose-limiting toxicity occurred with reversible grade 3 liver toxicity (increase of AST, ALT, AP, or γ-GT), hypokalaemia and fatigue in altogether five patients (one of these also at a rML-dose of 4.8 μg/kg) [20,21]. After the first injection of 5000 ng/kg rML, two VAE pre-exposed patients had an anaphylactic reaction with generalized urticaria; one of them also with dysphagia and swelling of the larynx [21]. Further grade 1-2 events included chills, diarrhoea and constipation, arthralgia, chest pain, tumour pain, headache, dizziness, flushing, itching, hypotension, anorexia, sweating, insomnia, mouth dryness, sensory neuropathy, taste disturbance [20-22]. One further grade 3 event was hypertension, occurring in 1 patient [22]. Further laboratory abnormalities documented during study period - it was unclear whether they were related to rML treatment or to advanced cancer disease or to other causes - were mostly grade 1-2, including thrombopenia (grade 1), anaemia, hypoalbuminaemia, hypoproteinaemia, hypo- and hypercalcaemia, hypo- and hypernatraemia, hypokalaemia, hyperbilirubinaemia, slight increase of creatinine [20-22].

Most of the ADRs or adverse events were reported from studies with no control group (37 studies, see Additional file 2), which impedes the discrimination of ADRs from underlying disease symptoms or spontaneously occurring events and therefore increases the risk of false positive findings; only part of these studies assessed causal relation with VAE or rML treatment. Of the 24 studies comparing outcome with a control group (see Additional file 2) and thus reducing the risk of bias, tolerability was usually good, without ADRs, except an angioedema/urticaria in one case, frequent LR (16 studies), fever, rise in temperature or FLS (5 studies), or individual cases of dry eyes, flatulence/loose stool and a "general vegetative reaction".

Altogether, most frequently reported ARDs in the studies were LR, i.e. erythema, induration, swelling, warmth and sometimes pain at the injection site; they were dose-dependent, self-limited and appeared less intensely and less frequently after some weeks of treatment. Histological investigation showed a superficial and deep, dense lymphoid infiltration in corium and subcutaneous fat: 60% T-cells (half CD4 and CD8 cells) and 40% macrophages. After intravenous infusion of VAE or rML in VAE-pre-exposed patients, LR sometimes appear at former injection sites. Also frequent, particularly after very high dosage, are FLS, fever, partly with chills, headache, irritation of the gastrointestinal system, pain (especially after intrapleural or intraperitoneal application), fatigue/dizziness or lassitude. Single, infrequent observations had been reversible liver toxicity (see above), mild infections and allergic reactions. The other reported ADRs were restricted to individual cases or to studies without comparison groups and without a clear dose-dependency or other observations to clearly distinguish the events from symptoms of underlying diseases.

### Animal experiments - characteristics

Regarding literature search and study selection see Figure 1 and Additional file 1. Altogether, 48 animal experiments or experimental settings matched inclusion criteria. Their characteristics are summarised in table 3 and details presented in Additional file 3. The quality of the experiments and their reporting, especially regarding immune outcomes and safety, varied substantially. Often, experiments consisted of several sub-experiments with safety results usually reported globally; accordingly, these sub-experiments are summarized in this review as well.

**Table 3:** Characteristics of the animal experiments (n = 48)

	Number of studies
**Animals**	
Mice	42
Rats (1 × mixed: rats & mice)	4
Horses, Cats	2

**Diagnoses**	
Healthy animals	21
Cancer * (2 × mixed)	27

**Treatment**	
Whole extract	34
Isolated or recombinant ML	14

**Application route**	
Subcutan, intraperitoneal **	35
Intratumoural, intravenous, intramuscular, intracutan, oral, intranasal, intravesical	13

**Application frequency**	
Applied just once	10
Applied more than once (5 × mixed) (up to several months)	38

**Maximum dose per application**	
≤ 20 mg mg/kg VAE	12
> 20-100 mg/kg VAE	16
> 100 mg/kg VAE (maximum dose: 1400 mg/kg)	6
> 50 ng/kg ML (maximum dose: 500 μg/kg oral, 50 μg/kg intranasal, 14 μg/kg sc)	9
< 50 ng/kg ML	5

**Observation time < 1 week**	**6**

**Treatment of control group**	
Placebo	33
No additional treatment or unclear	14
No control group	1

**Immune outcomes investigated**	**38**
Peripheral blood: CBC, DBC, leukocytes, lymphocytes, monocytes, granulocytes, T-cell subsets, activation markers, TNF-α	14
Immunoglobulins/humoral response to foreign antigens	9
Cellular response to foreign antigens, foreign skin graft rejection	2
Thymus (size, histological analysis, thymocytes, subsets, function	14
Spleen (size, weight, morphometric analysis, splenocytes, subsets, function)	8
Lymph nodes (weight, morphometric analysis)	2
Peritoneal macrophages and activity	3
Influence on leukopenia caused by radiation, chemotherapy, dexamethasone.	4
Others (tumour tissue, urinary bladder tissue, bronchoalveolar lavage)	4

**Safety outcomes investigated**	**17**
Monitored for toxicity, tolerability, vitality, clinical signs, body weight, food/water consumption, behavior, physical responses of the animals, local effects	9
Necropsy	2
Bladder histology after intravesical instillation of VAE	1
No details on recording	8

**Time schedule of safety assessment**	
Daily, 2/week	5
„Regular"	1
No details or no systematic plan	11

* therapeutic application of VAE in 2 studies: equine sarcoid in horses [92], fibrosarcoma in cats [93];

** Injection of tumour cells that had been pre-incubated with VAE or ML in 2 studies [98,99]

### Animal experiments - immunological results (see Additional file 3)

Most animal studies reported an increase or no change of the measured immune parameters and some a decrease of certain parameters.

*Incidence and duration of infections *were not measured in the reviewed studies.

*Haematology: *One study described a decrease of the *relative *amount of neutrophils and monocytes, 24 hours after single application of VAE (5 mg/kg), and a relative increase of lymphocytes. These were interpreted as immunostimulatory, however, no absolute counts were reported [81]. All other experiments observed no leukopenia or haematological toxicity or significant decrease of leukocytes. Leukocytes either increased or showed no difference to the control group, also after application of higher doses of VAE. Endoxan-, radiation- or dexamethasone (DX)-induced leukopenia was reduced by VAE.

*Unspecific serum immunoglobulins: *One study assessed serum proteins - total proteins, albumin, globulins α_2_, β and γ, composed by a multitude of different proteins - 24 hours after a single injection of VAE (5 mg/kg) and found lower levels of total proteins and the globulins than in a control group [81].

*Humoral or cellular immune response against foreign antigens *(immune function tests): All ten experiments showed a substantial enhancement of the immune response, but two also found varying results: In one study - insufficiently described - VAE was applied daily in three dosages (Isorel 14 or 140 or 1400 mg/kg) over 1 or 14 or 25 days in mice before or concomitant to immunisation with sheep red blood cells (SRBC), assessed by the plaque-forming cell (PFC) assay. The results varied substantially between all groups, also between the three placebo (control) groups, associated with a large standard deviation; most results showed an increase of PFC after VAE application, except a decrease after application of VAE over 5 weeks (25 injections), especially in lower dosage; this decrease had a similar magnitude as the variation between the three control groups, where PFC increased fivefold (and standard deviation more than thirtyfold) with repeated applications of saline; the cumulative dosage showed no dose-response pattern [82,83]. In the other study, VAE was given during 3 days either before or 6 days after the injection of SRBC in mice, and an either reduced or increased antibody titer was observed (day 10), respectively; the dose was comparatively low [84]. (Another similar experiment found an *increased *antibody formation when VAE was applied before, and no change when it was given after SRBC [85].) All other 8 experiments showed a substantial increase of humoral (antibodies, IgG, IgA, PFC) or cellular (T-cells, DTH) response after antigen stimulation in combination with VAE or ML, partly applied in very high doses [86-90].

*Immune organs: *Size and weight of thymus and spleen mostly increased and sometimes did not change; histology showed a hyperplasia of the thymus cortex and a proliferation of lymphoid cells in spleen and lymph nodes. Number and function (mitogen stimulated proliferation, secretion of cytokines, NK-cytotoxicity, ADCC) of splenocytes and thymocytes and their subsets also increased or stayed unchanged after application of VAE or ML (sometimes in high dosage), except in one study that found, 48 hours after injection of 30 ng/kg recombinant ML I, a decrease of splenocytes' NK-cytotoxicity (and of the number of circulating LGLs), while they increased after injection of 0.5 or 1 ng/kg rML and stayed unchanged after 3 and 10 ng/kg; also lymphocytes increased, while polymorphonuclear leukocyte and lymphocyte subsets showed no change [91].

*Lymphocytes *showed increased levels in the peripheral blood after VAE or ML application.

Remaining immune parameters - i.e. *number or cytotoxicity of peritoneal macrophages, macrophages in bronchoalveolar lavage: *The studies showed no depression but either increase or no change.

Altogether, no immunosuppression caused by VAE or ML was found in any of the studies, not even when high dosages of VAE of ML were applied.

### Animal experiments - ADRs (see Additional file 3)

Ten of the 17 studies, including the two therapeutic trials [92,93], reported good tolerability and no ADRs except mild edema at the injection site in a RCT on horses. One study reported weight loss at 50 mg/kg of a lectin-rich VAE preparation, good tolerability and no toxicity of the other VAE preparation up to 100 mg/kg [94]; one further study reported local intolerance after intravesical application of high doses of VAE (12000 ng ML/kg) [95]. Altogether five studies observed increasing toxicity and lethality when applying very high doses [42,96-99], close to or higher than LD_50 _(median lethal dose) [12,33,98,99] and far exceeding doses normally used, so these lethal events are more a matter of poisoning [12].

## Discussion

In the 69 clinical and 48 animal studies reviewed, assessing a variety of immune parameters and ADRs, no indication for an immunosuppressive effect through higher dose VAE or ML application was found. The studies reported stable, fluctuating or increased immune outcomes. Reported ADRs mainly consist of dose dependent FLS, local reactions at the injection site, fever and chills (especially under higher dose), also headache, fatigue and mild gastrointestinal symptoms. In a few cases, after intravenous infusion of very high doses of recombinantly produced ML (4800 and 6400 ng/kg), reversible hepatotoxicity and anaphylactic reactions were observed. Angioedema/urticaria was also observed in 2 other patients [32,34], one of which occurred only after the application of higher dosage VAE, and was no longer observed after dose reduction. Of all animal experiments five reported lethality after application of very high doses [42,96-99], within the dosing range of lethal doses [33,34], e.g. in an experiment published in 1987: 200 ng (10000 ng/kg) ML I given intraperitoneally in mice [97].

These observations are in accordance with the excluded studies which were also screened and which, in general, showed good tolerability and comparable ADRs. Also, previous analyses of ADRs and toxicity had similar results: An HTA-report analysing all clinical studies on VAE and all case reports did not find organ toxicity or biochemical changes, but frequent reporting of self-limited, dose-dependent LR and FLS and the occasional occurrence of allergic or pseudoallergic reactions [34]. The latter are also described in published case reports [32,34]. Additionally, one case of sarcoidosis potentially induced by VAE has been published [100]; observational studies report a favourable outcome after VAE application in sarcoidosis [101,102]. One review asked health authorities for reported adverse events connected to VAE treatment [31]; here, however, causal relationship between the reported adverse events and the VAE application had not been investigated and is questionable, especially as in at least some cases the adverse events are well-known symptoms of the underlying disease or the side effects of co-medications. Toxicologic investigations were reviewed as well; no indications for chronic toxicity were found [12,33]. Still, mistletoe - perhaps influenced by its unfortunate reputation as "kiss of death" and having been misused for killing the god Baldur in Norse mythology - gave rise to a number of reports in the popular and medical literature on a variety of alleged harm. These, however, did not have any or only a faulty empirical basis [34]. Also an acute "mistletoe hepatitis" was reported to have occurred after oral consumption of herbal tablets [103] which, however, were subsequently shown not to have contained any VAE [104]. In rats, VAE prevented acute hepatic damage caused by carbon tetrachloride [105].

Regarding immune parameters, a few of the reviewed studies reported decreases of outcomes. "Downs" of immune parameters, however, do not necessarily document an immunosuppression: Immune cells and molecules fluctuate naturally in time and vary between individuals. Also daily activities, diseases, the way of taking blood samples and its analysis have a major impact [12,55,57,58]. Hence ups and downs as well as statistical regression to the mean (decrease of initially high values, increase of initially low values) have to be expected. Furthermore, measurements from the peripheral blood mirror only a secondary compartment of the immune system as the relevant functions take place directly in the lymphoid or tumour or inflamed tissue. Besides their high turnover, immune cells fluctuate and only a minor portion is in the peripheral blood - for instance 2% of lymphocytes, while 98% are in tissues. Redistribution of cells towards tissue with - transient - decrease in the blood compartment is not indicative of immunosuppression [12,57,59,106-108]. Last but not least, the immune system functions as a network with manifold modifying, synergistic, antagonistic, redundant, context-dependent interactions of the pluripotent, pleiotropic and multifunctional cytokines and cells. Within this network quantitative changes of single parameters have a different meaning than within an isolating assay, and their clinical interpretation is arbitrary to a large extent. More important is therefore the performance of the complete immune system - for instance susceptibility to infectious disease or to cancer or functional tests, i.e. immune response to foreign antigens - and in the case of laboratory measurements, whether essential immune parameters (CBC, DBC, immunoglobulins) can show a clear pathological aberration.

In the reviewed studies, number and duration of infections decreased in immunocompromised children as well as in healthy subjects but showed an increase in HIV positive patients: transient exacerbations of gingivitis, candidiasis, FLS, sinusitis and herpes simplex in a clearly dose-dependent manner [63,64]. This increase, however, was neither due to disease progression, nor accompanied by suppression of immune parameters [63,64,109] and may have been caused by an increase of the inflammatory response due to immunostimulation through VAE. Nevertheless, these observations should be investigated in more detail. Furthermore, in these studies VAE had been applied in comparatively low doses. Studies applying high doses did not explicitly mention clinical infections, but as they mostly closely monitored patients for ADRs a potential increase of infections or infectious complications should have been noticed. Two pharmacoepidemiologic studies - excluded here as the applied dosage was not reported - found no difference in infections and mucositis in VAE-treated cancer patients compared to the control group [110,111].

Other functional assessments of the immune system refer to the humoral and cellular response to foreign antigens in a variety of animal experiments. They showed a substantial enhancement of immune response, even when ML were applied in very high dosages (see Additional file 3). Differing results in subgroups of two of the 10 experiments [82-84] are most likely caused by experimental variation, as the outcome in control groups varied to a similar amount and as the applied methodology - the SRBC and PFC assay - is known to be susceptible to variable results due to subjectivity in measurement, low automation and limited stability of SRBC [112,113].

Most studies analysed parameters in the peripheral blood which allows only indirect conclusions; one study, however, examined tumour tissue, immunologically a highly relevant compartment. High dosages of VAE had been injected intratumourally (up to 42000 ng ML). No immunosuppressant effects were seen but an increase of some relevant immune cells [114]. Similar results were observed in animals, where tumour tissue and immune organs were analysed repeatedly (see Additional file 3).

In the reviewed studies and experiments, no pathological laboratory immunosupression was observed. Decreases were largely within the normal range and often with parameters whose downs as well as ups could both be considered favourable: For instance, an exploratory study (without a control group to differentiate from natural course and from influence of cancer disease) had observed a small decrease of CD3+T-cells expressing CD25 after mitogen (PHA) stimulation ("activated" T-cells) during 6 month VAE treatment in cancer patients; this decrease had been more pronounced in the group with initial higher baseline values, which was higher than normal controls [75] - a pattern typical for a mere statistical regression. Here, "downs" as well as "ups" of the CD3+T-cells expressing CD25 can be clinically interpreted in four directions: 1) spontaneous variation without major clinical relevance (statistical regression); 2) "normalisation" (towards normal values); 3) "suppression" (CD25+T-cells interpreted as "helper cells"); 4) "stimulation" (CD25+T-cells as containing CD4+CD25+ T-cells that comprise *regulatory T-cells*, which suppress an effective immune response against tumours [115-117]).

When applying active substances, especially in high dosages, acute, stress-related changes of immune parameters can be observed. In contrast to chronic stress, which suppresses or disrupts immune functions, this acute stress often acts as an immunoenhancer. Probably mediated by glucocorticoid and catecholamine hormones, acute stress significantly changes leukocyte distribution and trafficking of dendritic cells, macrophages and lymphocytes in the body. This leads to changes in the number and composition of leukocytes which can be seen as a transient increase or decrease of the number of certain immune cells (e.g. lymphocytes, T-, B-cells, NK-cells, monocytes, neutrophils) and their functional capacity (proliferation, cytotoxicity, phagocytosis, antibody production, etc.). These changes are rapidly reversed again and can differ in animals and humans [60].

These short-term changes are also observed after VAE or ML application, when immune parameters are measured within hours or days: 6 hours after VAE application a decrease of NK- and ADCC activity and of the numbers of lymphocytes, LGLs and monocytes was measured, while neutrophils increased. 24 hours later the cell numbers and activities had normalised or increased [76,77,118,119]. After application of recombinant ML, a slightly different pattern was observed: a decrease of splenocytes' NK-cytotoxicity and of the number of circulating LGLs was observed 48 hours after injection, while lymphocytes increased and neutrophils showed no change [91]. These differences may be influenced by spontaneous or experimental variation but may also be attributed to the application of recombinant ML, whose biological and biochemical effects differ from natural ML due to lack of glycosylation [12,120]. In contrast, after application of VAE and isolated peptides, an increase in NK-cytotoxicity was observed after 2 - 4 days [121].

This review concentrates on VAE and ML (ML I, II and III). ML are important and biologically active compounds of VAE, but they are not the only relevant compounds (see background section). The other compounds, however, are less thoroughly investigated, and little data is available in animals and humans.

ML content of different preparations cannot be compared and can differ also for the same preparation (see methods section). Therefore, the information presented here serves only as a rough orientation. Furthermore, one should bear in mind that there are many further animal experiments conducted on VAE, ML or VAE compounds than those presented here (Figure 1); most of these experiments examine antitumour effects and should not be missed if an according objective is addressed; our review, however, focused on immune outcomes and ADRs and therefore included only a small part of these experiments.

This review is the first to systematically and comprehensively assess all animal and human studies on potential side effects of therapeutically applied high-dose VAE or ML. The question arises whether a bias is inherent as all study designs were included and as several of the included studies have minor or major methodological weaknesses. For instance, some of the studies had insufficiently described methods for detecting adverse effects or scantily reported the results. This is important as different methods of monitoring for adverse effects yield different results. For instance, active surveillance yields higher frequencies than passive, less-formalised methods [122]. Also reporting, definition, combined reporting or generic statements influence the results [49]. Still, most of the clinical studies, and especially all RCTs and all studies with safety as primary objective had reported standardised assessment of immune parameters according to existing guidelines. Single-arm studies, comparing immune outcomes with the status before intervention, have a high risk of bias, i.e. of false positive findings: the changes often cannot be differentiated from effects of other factors that influence the immune system and may induce adverse events; these factors are omnipresent in cancer patients: cancer itself, anticancer treatment, cancer remission, other medications, complications, infections, etc. We therefore consider false positive results - i.e. adverse events or immune "depressions" that were in fact not caused by VAE or ML - to be highly probable, especially in single-arm studies. Even when healthy young subjects are asked to document everyday symptoms for a couple of days, the occurrence of common unspecific "side effects" have a similar magnitude as in clinical studies, i.e. fatigue in 41% or headache in 15% [123]. Nevertheless, we have included studies rather widely. Single-arm studies investigated higher dosages and alternative routes of application, and observational studies better reflect actual patient care. We did not want to miss potentially relevant risks of VAE treatment and consider it less problematic to overreport unspecific side effects than to miss a relevant major risk. Altogether, the results are coherent and the kind of ADRs in principle plausible - although they might be overrated, especially in studies without a proper control group.

The studies may have missed ADRs that were too weak or short to be documented in the studies or linked to very high dosages. However, as the studies reviewed had investigated a large range of doses as well as all relevant application forms, as the treatment and screening periods covered different periods - daily, weekly, monthly -, and as relevant recommended parameters had been documented and as patients with different diseases and disease-related or therapy-induced organ dysfunction had been included, we consider the unnoticed occurrence of major and clinically relevant ADRs to be highly unlikely. Minor, less relevant or short term effects or very rare events, however, may have been missed.

In order to minimize publication bias in this review, a comprehensive search was conducted, unpublished trials were included and we had long discussions with experts in the fields. We consider it unlikely that important, rigorous trials went unnoticed, at least in Europe. However, we cannot exclude the possibility that we missed minor ones, or trials conducted in distant, non-European countries. Furthermore, studies and especially animal experiments did not report all details on immunological parameters and safety outcomes; we presume that all relevant data is presented but we could not verify this issue.

In total, VAE seem to exhibit a low risk also in higher dosages, but, depending on the dosage, dosage change, application form and individual tolerability, the patient should be monitored or the treatment directly applied by the physician. Regarding research, future clinical studies should follow general recommendations for assessing and reporting harm (e.g. [124]) and adverse event case reports should take into account corresponding guidelines for publication (e.g. [125]). Reports on animal experiments - that only rarely mentioned adverse effects - should, in the future, include a description of tolerability and harm of VAE and its compounds.

## Conclusions

In 48 animal experiments and 69 clinical studies investigating higher dosages of VAE, no indications of immunosuppression were found. Quite the opposite, most of the studies describe a distinct immunostimulation, even at high dosages of VAE or ML. As the investigations comprised high doses, relevant treatment and assessment periods and relevant parameters, we consider clinically relevant immunosuppression to be unlikely. ADRs observed during higher dose VAE or ML often consist of LR and FLS, or some non-specific effects, and allergic or pseudoallergic reactions in some cases, and, after application of high-dose recombinant ML, of reversible hepatotoxic effects. For normal application VAE is safe: it can also be presumed to be of low risk when used for local or systemic high dose application if it is monitored by an experienced physician.

## Competing interests

Within the last five years IFAEMM has received restricted research grants from the pharmaceutical companies Wala, Weleda, Abnoba, and Helixor, who produce mistletoe medications.

## Authors' contributions

GSK and HK designed the review, read and assessed all studies. GSK and RG conducted the search, checked inclusion criteria, extracted the data. GSK conducted the analysis which was checked and critically discussed with HK and RG. All authors read and approved the final manuscript.

## Pre-publication history

The pre-publication history for this paper can be accessed here:

http://www.biomedcentral.com/1472-6882/11/72/prepub

## Supplementary Material

Additional File 1 **Literature search**. All databases, search terms, full electronic search strategy for all databases, including limits used, and detailed results for all databases. Click here for file

Additional file 2 **Controlled clinical studies investigating Viscum album > 1 mg or ML > 1 ng/kg body weight: 1) Comparing immune and safety outcome with a control group; 2) Comparing immune and safety outcome pre-treatment and post treatment**. Characteristics for each clinical study included in the review: diagnosis, study size, preparation, application, dosage per application, treatment (follow-up) period, concomitant therapy, immune and safety parameter investigated, compartment investigated, assessing frequency (safety parameter), immune outcomes compared to control group or to pre-treatment, safety outcome, and citation. Click here for file

Additional file 3 **Animal experiments investigating Viscum album > 0.02 mg/kg or ML > 1 ng/kg body weight and comparing immune and safety outcomes with a control group**. Characteristics for each animal experiment included in the review: animals, diagnosis, preparation, application, dosage per application, treatment (follow-up) period, concomitant therapy, immune and safety parameter investigated, compartment investigated, assessing frequency (safety parameter), immune outcomes compared to control group, safety outcome, and citation. Click here for file
