# Supplementary material for: Safety of higher dosages of Viscum album L. in animals and humans - systematic review of immune changes and safety parameters
Source: BMC Complement Altern Med. 2011 Aug 28;11:72. doi: 10.1186/1472-6882-11-72 (PMC3180269; doi:10.1186/1472-6882-11-72)
Supplement: Additional File 1 — Literature search. All databases, search terms, full electronic search strategy for all databases, including limits used, and detailed results for all databases. [file 1472-6882-11-72-S1.PDF]

Additional File 1: Literature Search

I. AMED, Biosis Previews, Embase, Medline/Premedline

```
SYSTEM:OS  - DIALOG OneSearch
  File 164:Allied & Complementary Medicine 1984-2011
         (c) 2011 BLHCIS
  File   5:Biosis Previews(R) 1926-2011
         (c) 2011 The Thomson Corporation
  File  73:EMBASE 1974-2011
         (c) 2011 Elsevier B.V.
*File  73: The 2011 Thesaurus has been installed with UD20110407.
  File 155:MEDLINE(R) 1950-2011
         (c) format only 2011 Dialog
*File 155: Medline has been reloaded with the 2011 MeSH
thesaurus.

       Set  Items  Description
       ---  -----  -----------
```

? s **MISTLETOE OR VISCUM? OR MISTEL? OR ISCADOR? OR ISCAR OR HELIXOR OR ABNOBA? OR ISCUCIN OR ISOREL OR VISOREL OR ?SOREL OR WELEDA OR WALA OR EURIXOR OR LEKTINOL OR PLENOSOL OR AVISCUMINE**

```
              4280  MISTLETOE
              4048  VISCUM?
               582  MISTEL?
               673  ISCADOR?
                 9  ISCAR
               171  HELIXOR
                85  ABNOBA?
                31  ISCUCIN
                63  ISOREL
                 0  VISOREL
                63  ?SOREL
               127  WELEDA
                81  WALA
                68  EURIXOR
                47  LEKTINOL
                58  PLENOSOL
                31  AVISCUMINE
       S1     6424  MISTLETOE OR VISCUM? OR MISTEL? OR ISCADOR? OR ISCAR OR
                    HELIXOR OR ABNOBA? OR ISCUCIN OR ISOREL OR VISOREL OR
                    ?SOREL OR WELEDA OR WALA OR EURIXOR OR LEKTINOL OR
                    PLENOSOL OR AVISCUMINE
```

? s **STUDY? OR STUDIE? OR TRIAL OR EVALUAT? OR RANDOM? OR INVESTIG? OR COHORT? OR KOHORT? OR OUTCOME? OR MOUSE OR MICE OR MAUS OR MAEUSE OR RAT? OR RATTE? OR RABBIT? OR KANINCHEN OR HORSE? OR PFERD OR SIDE(w)EFFECT OR ADVERSE(w)EFFECT OR ADVERSE(w)DRUG(w)REACTION OR TOXICITY OR COMPLICATION? OR NEBENWIRKUNG OR VERTRAEGLICHKEIT**

```
          15610390  STUDY?
          10983155  STUDIE?
           1551481  TRIAL
           5407543  EVALUAT?
           1752541  RANDOM?
```

```
  5520122  INVESTIG?
   568336  COHORT?
      413  KOHORT?
  2619397  OUTCOME?
  2621073  MOUSE
  2222820  MICE
     2902  MAUS
       29  MAEUSE
 12123351  RAT?
    18415  RATTE?
  1041316  RABBIT?
     2963  KANINCHEN
   268610  HORSE?
     1109  PFERD
  1528065  SIDE
  7482285  EFFECT
   641133  SIDE(W)EFFECT
  2679352  ADVERSE
  7482285  EFFECT
    55679  ADVERSE(W)EFFECT
  2679352  ADVERSE
 13734686  DRUG
  3749774  REACTION
  1041368  ADVERSE(W)DRUG(W)REACTION
  1561938  TOXICITY
  4088940  COMPLICATION?
      425  NEBENWIRKUNG
       37  VERTRAEGLICHKEIT
S236169596  STUDY? OR STUDIE? OR TRIAL OR EVALUAT? OR RANDOM? OR
            INVESTIG? OR COHORT? OR KOHORT? OR OUTCOME? OR MOUSE OR
            MICE OR MAUS OR MAEUSE OR RAT? OR RATTE? OR RABBIT? OR
            KANINCHEN OR HORSE? OR PFERD OR SIDE(W)EFFECT OR
            ADVERSE(W)EFFECT OR ADVERSE(W)DRUG(W)REACTION OR TOXICITY
            OR COMPLICATION? OR NEBENWIRKUNG OR VERTRAEGLICHKEIT
```

? **s s1 AND s2**

```
     6424  S1
 36169596  S2
S3    4233  S1 AND S2
```

? **rd s3**

```
S4    2879  RD S3  (unique items)
```

References found: 2879

II. Cochrane Library

(Cochrane Database of Systematic Reviews, Cochrane Controlled Trials Register, The NHS Economic Evaluation Database, HTA Database), clinical trials:

MISTLETOE OR VISCUM OR MISTEL OR ISCADOR OR ISCAR OR HELIXOR OR ABNOBA OR ISCUCIN OR ISOREL OR VISOREL OR WELEDA OR WALA OR EURIXOR OR LEKTINOL OR PLENOSOL OR AVISCUMINE

References found (no exclusions): 70

III. NLM Gateway

(Medline already included in other search):

(1317	**MEDLINE/PubMed** - journal citations, abstracts)
57	**NLM Catalog** - books, AVs, serials
13	**Bookshelf** - full text biomedical books
239	**TOXLINE Subset** - toxicology citations
0	**DART** - Developmental and Reproductive Toxicology
7	**Meeting Abstracts**
3	**MedlinePlus** - Health Topics
0	**MedlinePlus** - Drug Information
1	**MedlinePlus** - Medical Encyclopedia
0	**MedlinePlus** - Current Health News
0	**MedlinePlus** - Other Resources
13	**ClinicalTrials.gov**
0	**DIRLINE** - Directory of Health Organizations
0	**Genetics Home Reference**
0	**Household Products Database**
1	**Images from the History of Medicine**
0	**HSRProj** - Health Services Research Projects
0	**OMIM** - Online Mendelian Inheritance in Man
3	**HSDB** - Hazardous Substances Data Bank
0	**IRIS** - Integrated Risk Information System
0	**ITER** - International Toxicity Estimates for Risk
0	**GENE-TOX** - Genetic Toxicology (Mutagenicity)
2	**CCRIS** - Chemical Carcinogenesis Research Information System
0	**Profiles in Science**

References found (no exclusions): 339

IV. Dissonline, **fulltext search:**

MISTLETOE: 96

VISCUM: 122

MISTEL: 88

ISCADOR: 31

ISCAR: 4

HELIXOR: 11

ABNOBA: 9

ISCUCIN: 3

ISOREL: 5

VISOREL: 0

EURIXOR: 0

LEKTINOL: 7

PLENOSOL: 3

AVISCUMINE: 0

References found (no exclusions): 379

V. Private databases

Keyword: MISTLETOE OR MISTEL

Title: MISTLETOE OR VISCUM OR MISTEL OR ISCADOR OR ISCAR OR HELIXOR OR ABNOBA OR ISCUCIN OR ISOREL OR EURIXOR OR LEKTINOL OR PLENOSOL OR AVISCUMINE

References found (no exclusions): 1970
