# Supplementary material for: Safety of higher dosages of Viscum album L. in animals and humans - systematic review of immune changes and safety parameters
Source: BMC Complement Altern Med. 2011 Aug 28;11:72. doi: 10.1186/1472-6882-11-72 (PMC3180269; doi:10.1186/1472-6882-11-72)
Supplement: Additional file 2 — Controlled clinical studies investigating Viscum album > 1 mg or ML > 1 ng/kg body weight: 1) Comparing immune and safety outcome with a control group; 2) Comparing immune and safety outcome pre-treatment and post treatment. Characteristics for each clinical study included in the review: diagnosis, study size, preparation, application, dosage per application, treatment (follow-up) period, concomitant therapy, immune and safety parameter investigated, compartment investigated, assessing frequency (safety parameter), immune outcomes compared to control group or to pre-treatment, safety outcome, and citation. [file 1472-6882-11-72-S2.PDF]

Additional file 2

Controlled clinical studies investigating Viscum album > 1 mg or ML > 1 ng/kg body weight: Comparing immune and safety outcome with a control group treated by placebo, etoposide, lentinan, multivitamins, Skin Control Test or no preparation.

Caution: ML are only one of several oncologically active ingredients. ML contents of VAE were obtained from the respective publication (*) or from the manufacturer or were calculated from another publication on the same preparation. They are often imprecise, as they are, for instance, based on average calculation of batches from several years. ML and Viscum album dosages from different preparations can only be compared when taking into account the different test systems (e.g. ELLA, ELISA) and the different extraction procedures (leading to different ML I, II, and III contents) used.

**Author, reference**	**Diag-nosis**	**Preparation, application**	**Dosage per application**		**Stu-dy size**	**Treatment (follow-up) period**	**Conco-mitant therapy**	**Immune parameter investigated**<br>*Compartment investigated*<br>Safety: Assessing frequency and method	**Immune outcome comp-ared to control group**<br>↑ above, ↓ below,<br>↔ no difference	**Safety outcome** (including "possible" relation)
			**Viscum album mg**	**ML ng**						
**RCTs – double- or single-blinded; participants either healthy or with cancer**										
Huber 2009	Healthy	Iscucin P, Viscum M, sc, 2/wk	up to 50 or 20		71	12 (17) wk	no	*Peripheral blood:* DBC, lymphocyte subsets, IL-6<br>Safety: Weekly controls	↑ Eosinophils, T-helper cells<br>↔ IL-6	No deviations in lab. parameters; LR
Huber 2006	Healthy	Iscador Qu sp. or P, sc, 2/wk	up to 5 or 20 resp.	up to 411 or 22 resp.*	47	3 (6) mth	no	*Peripheral blood:* PMBC: IQ- or IP-induced proliferation, release of IFN-γ, TNF-β, IL-5, IL-13, IL-1, TNF-α<br>Safety: No details	↑ IQ: Proliferation, TNF-α, INF-γ IP: Proliferation<br>↓ IP: IL-5, IL-13	No severe adverse reactions; LR
Huber 2005/06	Healthy	Iscador Qu sp., ML-depleted, or ML, sc, 2/wk	up to 5	up to 428, 22 or 401 resp.*	43	8 wk	no	*Peripheral blood:* DBC, VAE-induced proliferation of lymphocytes, release of GM-CSF, IFNγ, IL-5; soluble IL-2 receptor<br>Safety: Biweekly tolerability (Likert scale), lab. parameters	↑ Leukocytes, granulocytes, eosinophils, GM-CSF, IFNγ, IL-5, soluble IL-2 receptor, proliferation of lymphocytes	No deviations in lab. parameters; moderate, good and excellent tolerability
Auerbach 2005	Breast Ca.	Helixor A, sc, 3/wk	up to 100	up to 248*	23	up to 6 (12) mth	CMF, RT	*Peripheral blood:* Leukocytes, CRP, lymphocyte subsets<br>Safety: Each cycle lab. parameters	↑ CD56+/CD69+/CD45<br>↔ Other parameters	Good tolerability; LR; headache

Author, reference	Diag-nosis	Preparation, application	Dosage per application: Viscum album mg	Dosage per application: ML ng	Stu-dy size	Treatment (follow-up) period	Conco-mitant therapy	Immune parameter investigated<br>*Compartment investigated*<br>Safety: Assessing frequency and method	Immune outcome comp-ared to control group<br>↑ above, ↓ below,<br>↔ no difference	Safety outcome (including "possible" relation)
Huber 2001/02	Healthy	Iscador Qu sp, P, sc, 2/wk	up to 5 or 20	411 or 22 resp.*	48	3 (6) mth	no	*Peripheral blood:* DBC, haptoglobin, CRP<br>*Clinical:* Common cold<br>Safety: Weekly: Side effects, patient-reported tolerability, lab. parameters, autoantibodies wk 6, 12, 24	↑ Eosinophils, haptoglobin<br>↓ common cold (trend)<br>↔ CRP, leukocytes, lymphocytes, neutrophils, monocytes	No severe ADRs; no deviations in lab. parameters; LR, FLS, dry eyes, flatulence/loose stool
Klein 2002	Healthy	Iscador Qu or P, sc, 2/wk	up to 5 or 20		47	12 (25) wk	no	Safety: No details		No severe ADRs, LR
Wispler 2001/05	Healthy	Iscador QuFrF, P, sc, 2/wk	up to 1 or 10 resp.	up to 200*	29	7 wk	no	*Peripheral blood:* DBC; Lymphocyte subsets: CD3/CD4/CD45RO, CD3/CD8/CD45RO, CD3/CD4/CD45RO/CLA, CD3/CD8/CD45RO/CLA and others; CRP<br>Safety: Regular: Lab. parameter, AE, sAE, organ functions, LR	↑ CD45RO+ CD4 and CD8, CD3, CD3/CD8, CD3/CD8/CD11a, CD3/CD8/HLA-DR, lymphocytes, eosinophils, leukocytes, monocytes, neutrophils	No sAE; no deviations in lab. Parameters; LR
**RCTs – not blinded; participants with cancer**										
Son 2010	Breast Ca.	Helixor, sc, 3/wk	up to 100		20	7 (8) wk	CAF, RT	*Peripheral blood:* IL-2, IL-4, IL-6, IL-10, TGF-ß, IFN-γ<br>Safety: No details	↑ IL-6, IFN-γ<br>↔ IL-2, IL-4, IL-10, TGF-ß	LR, fever
Hekal 2009	Bladder Ca.	Abnobaviscum Fr, intravesical (6 wk) then sc, 1/wk	100 and, 40 resp.	50000 and 20000 resp.*	60	1 yr	(control: BCG)	Safety: Quarterly: Side effects, lab. parameter, cystoskopy		No severe toxicity; no lab. changes; LR; safe, tolerable
Longhi 2009	Osteo-sarcoma	Iscador P, sc, 3/wk	up to 20		10	12 mth	(control: Etoposide, G-CSF)	*Peripheral blood:* CBC, lymphocytes, T-cells, NK cells, CD4, CD8, IL-2, IL-4, IL-12, IL-15, IFN-γ, IP10<br>Safety: Monthly, quarterly: Toxicity according to WHO criteria	↑ T-lymphocytes, NK-cells; (IFN-γ, IP10 in subgroup)	No toxicity; LR
Tröger 2009	Breast Ca.	Iscador M sp., sc, 3/wk	up to 5	up to 290	61	6 mth	CAF	*Peripheral blood:* CBC, DBC, lymphocytes (CD4, CD8, NK-, B-cells, HLA-DR)<br>Safety: Each cycle: Toxicity according to CTC, AE, sAE (interview), LR (diary)	↓ CAF-induced neutropenia<br>↔ Immunological parameters	LR, no other VAE-related AE

Author, reference	Diag-nosis	Preparation, application	Dosage per application<br>Viscum album mg	 ML ng	Stu-dy size	Treatment (follow-up) period	Conco-mitant therapy	Immune parameter investigated<br>*Compartment investigated*<br><u>Safety:</u> Assessing frequency and method	Immune outcome comp-ared to control group<br>↑ above, ↓ below,<br>↔ no difference	Safety outcome (including "possible" relation)
Büssing 2008	Breast Ca.	Iscador M sp., iv, 2/cycle	up to 5	up to 290	65	day 8 of cycle	(F)EC, G-CSF (partly)	*Peripheral blood:* PMA-, E.coli-stimulated burst of granulocytes, lymphocyte subsets<br><u>Safety:</u> No details	↔ Granulocyte function, lymphocyte subsets	Safe, tolerable
Tröger 2008	Breast Ca.	Helixor A, sc, 3/wk	up to 200	up to 400 – 1400	65	6 mth	CAF	*Peripheral blood:* CBC, DBC, lymphocytes (CD4, CD8, NK-, B-cells, HLA-DR)<br><u>Safety:</u> Each cycle: Toxicity according to CTC, AE, sAE (interview), LR (diary)	↓ CAF-induced neutropenia<br>↔ Immunological parameters	LR; no other VAE-related AE
Schink 2007	Colorec-tal Ca.	Iscador M sp., iv, once	5	up to 290	22	(7) d	surgery	*Peripheral blood:* DBC, NK cell activity, HLA-DR on monocytes, CRP<br><u>Safety:</u> Regular lab. parameter; AE	↓ Surgery-induced suppression of NK cell activity<br>↔ HLA-DR on monocytes	No VAE-related AE
Cazacu 2006	Colorec-tal Ca.	Isorel A, iv, daily	300		40	10 preope-rative (+ 10) d	surgery	*Peripheral blood:* TNF-α<br>*Tumour tissue:* Peritumoural infiltration, tumour infiltrating lymphocytes<br><u>Safety:</u> VAE-related AE, CBC	↑ TNF-α, tumoural lymphocytic infiltrate	No VAE-related AE
Enesel 2005	Digestive tract Ca.	Isorel A, sc, 3/wk	60, 120 or 180		70	4 wk	surgery	*Peripheral blood:* Leukocytes, lymphocytes and subsets (CD2, CD3, CD19, CD4, CD8, NK cells), IgG, IgA, IgM, C3, C4	↑ Lymphocytes, T-, B-, NK cells, CD4, C3, C4, IgA, IgM, IgG<br>↓ CD8<br>↔ Leukocytes	
Piao 2004, Klose 2003	Breast, ovarian, lung Ca.	Helixor A, sc, 3/wk	up to 200	up to 400 – 1400	233	up to 8 wk	CT	*Peripheral blood:* DBC, CD3, CD4, CD8, NK cell activity<br><u>Safety:</u> Toxicity according to WHO criteria, lab. parameters, AE	↔ Immune outcomes	LR, angioedema/ urticaria, fever, no other ADRs
Cazacu 2003	Colorec-tal Ca.	Isorel A, iv, 3/wk	300		64	ca. 6 mth	surgery, 5-FU	<u>Safety</u>: Quarterly: Clinical examination, lab. parameters		No VAE-related AE
Dold 1991	Lung Ca.	Iscador U or Qu, sc, 3/wk	up to 30		227	unlimited (> 2 yr)	no	*Peripheral blood:* Leukocytes<br><u>Safety:</u> Monthly: Clinical examination, lab. parameters; patient-diary	↔ Leukocytes	LR, fever; no lab. changes
Gutsch 1988	Breast Ca.	Helixor, sc, 3/wk	up to 30		643	6 yr	surgery, RT	<u>Safety</u>: Quarterly follow-up; no systematic assessment		LR, rise in temperature; no organ toxicity
Douwes 1986	Colorec-tal Ca.	Helixor, sc, daily	up to 200		60	ca. 1 yr, no exact data	5-FU, folinic acid	<u>Safety:</u> No details		No VAE-related AE

Author, reference	Diag-nosis	Preparation, application	Dosage per application: Viscum album mg	Dosage per application: ML ng	Stu-dy size	Treatment (follow-up) period	Conco-mitant therapy	Immune parameter investigated<br>*Compartment investigated*<br>Safety: Assessing frequency and method		Immune outcome comp-ared to control group<br>↑ above, ↓ below,<br>↔ no difference	Safety outcome (including "possible" relation)
Lange 1985	Lung, ovarian, ENT Ca.	Helixor A, sc	up to 200	up to 1400	68	8-12 wk	CT, RT	*Peripheral blood:* Leukocytes<br>Safety: During CT: Daily toxicity (Lilly tables), weekly lab. parameters	↑<br>↔	Recovery from leukopenia<br>Leukopenia	No VAE-related AE, no lab. changes
**Non-RCTs; participants with cancer**											
Loewe-Mesch 2008	Breast Ca.	Iscador M sp., sc, daily – 2/wk	up to 2.5	up to 125*	66	3 mth	CMF/EC; others: more in control group	*Peripheral blood:* DBC, lymphocyte subsets, mitogen stimulation of lymphocytes<br>Safety: Patient diary; every cycle AE	↑<br>↓<br>↔	Eosinophils<br>Lymphocytes (more with glucocorticoids)<br>Immune parameter	LR, no VAE-related sAE
Douwes 1988	Colon Ca.	Helixor, sc, 3/wk	100-200		40	Months (no data)	5-FU, folinic acid	*Peripheral blood:* CBC<br>Safety: Monthly: Lab. parameter		No leukopenia	No data
Fellmer 1966	Cervical Ca.	Iscador M, sc, 2-3/wk	up to 20		790	up to 3 yr (5 yr)	no	Safety: No details			Well tolerated, LR; vegetative reaction

Clinical studies investigating Viscum album > 1 mg or ML > 1 ng/kg body weight: Comparing immune and safety outcome pre-treatment and post treatment

Author, reference	Diagnosis	Preparation, application	Dosage per application		Study size	Treatment (observation) time	Concomitant therapy	Immune parameter investigated<br>*Compartment investigated*<br>Safety: Assessing frequency and method	Immune outcome compared to pre-treatment<br>↑ increase, ↓ decrease, ↔ no change	Safety outcome
			Viscum album mg	ML ng						
**Healthy participants**										
Huber 2010		Abnobaviscum Fr, sc, once	20	20000*	15	(14 d)	no	*Peripheral blood:* CBC, NK cells (CD16/CD56; CD54/CD94), CRP<br>Safety: Regularly: Lab. parameter, AE	↔ CD54/CD94	LR, rise in temperature, FLS, nausea; no sAE; no deviation in lab parameter or ECG
Gorter 1998		Iscador QuFrF, Qu sp., sc, 3/9d	up to 2.5	up to 500 or 190 resp.*	7	9 (10) d	no	*Peripheral blood:* DBC, T- and B-lymphocytes, CD3/CD4, CD3/CD8, CD3/CD25, CD8/CD38<br>Safety: Once: LR, including histology	↑ Leukocytes, granulocytes, neutrophils, monocytes<br>↓ Lymphocytes, T-lymphocytes within normal range	LR
Stein 1998		VAE M, sc, 3/wk	up to 10	up to 2350*	8	8 (12) wk	no	*Peripheral blood:* Cytokines; proliferation & cytokine release of lymphocytes	↑ Proliferation (4 wk), TNF-α/IL-6	
Hajto 1989		Iscador QuFrF, sc, once	3.15	220*	4	(24 h)	no	*Peripheral blood:* Number and phagocytosis of granulocytes, LGL	↑ All parameters	
**Participants of each study partly healthy, partly with HIV or cancer**										
Gorter 1999	HIV	Iscador QuFrF, sc, 2/wk	up to 5	up to 550	41	4-15 (5-17) mth	AZT (partly)	*Peripheral blood:* CBC<br>*Clinical:* Infections<br>Safety: 2/wk, biweekly: AE, lab. parameter; according to WHO criteria	↑ Eosinophils, inflammatory diseases<br>↔ Other cells	LR, fever, FLS, fatigue, headache; slight ↑: Urea, creatinine, slight ↓ Albumin, HB (normal range)
Stoss 1999	HIV	Iscador Qu sp., QuFrF, sc, 2/wk	up to 5	175 or 550 resp.*	47	18 wk	no	*Peripheral blood:* CBC, CD3/25+, CD8/CD38+<br>Safety: Biweekly: Injection site	↑ Eosinophils, CD3/25+, CD8/38+<br>↔ Neutrophils	LR
Van Wely 1999	HIV	Iscador Qu sp., sc, 2/wk	up to 5	up to 175	24	6 mth	AZT, DDI, DDC (partly)	*Peripheral blood:* CBC<br>*Clinical:* Infections<br>Safety: Biweekly: AE, lab. parameters	↑ Eosinophils, inflammatory diseases<br>↔ Other cells	LR, FLS, fever, fatigue, headache; slight ↑: Urea, creatinine; slight ↓: Albumin, HB (normal range)
Stoss 1998	HIV	Iscador QuFrF, sc, 2/wk	up to 5	up to 550*	18	4 (5) mth	no	*Peripheral blood:* IFN-γ (serum)<br>Safety: No details	↔ IFN-γ	Well tolerated

Author, reference	Diagnosis	Preparation, application	Dosage per application: Viscum album mg	ML ng	Study size	Treatment (observation) time	Concomitant therapy	Immune parameter investigated *Compartment investigated* Safety: Assessing frequency and method	Immune outcome compared to pre-treatment ↑ increase, ↓ decrease, ↔ no change	Safety outcome
Gorter 1996	HIV, cancer	Iscador QuFrF, Qu sp., sc, 2/wk	up to 5	up to 525 or 350 resp.*	78	65 wk (6-15 mth)	AZT, DDI, DDC (partly)	*Peripheral blood:* DBC, CD4, CD4/CD8, CD3/25 lymphocytes<br>Safety: Biweekly lab. parameters	↑ CD3/25 lymphocytes, eosinophils<br>↓ Lymphocytes (initially transitional)	No drug-related AE
**Patients with localised or advanced cancer or other diseases**										
Eisenbraun 2011	Breast Ca.	Abnobaviscum M, sc, 3/wk	up to 2	up to 900*	130	5 mth	CT, HT, RT (partly)	*Peripheral blood:* Leukocytes, lymphocytes, eosinophils<br>Safety: Once: Patient- and physician-reported AE, tolerability (rating scale)	↑ Leukocytes, lymphocytes, eosinophils	LR, mild side effects (lassitude, headache, unspecific malaise, dizziness, dermatitis)
Ebrahim 2010	Liver Ca.	Abnobaviscum Fr, sc, 1/wk	40	20000*	120	2-121 wk	no	*Peripheral blood:* CBC<br>Safety: Monthly: Toxicity according to NCI CTC	No haematologic toxicity	Well tolerated; LR, fever
Mansky 2008/10	Various Ca.	Helixor A, sc, daily	up to 250	up to 500-1750	44	9 wk	Gemcitabine	*Peripheral blood:* Leukocytes, neutrophils; IL-6, IL-12, IFN-γ, TNF-α;<br>Safety: Closely monitored: Toxicity, according to CTC	↔ Cytokines<br>↑ Nadir of neutrophils (trend)	LR, fever, FLS; Cellulitis (grade 3)
Bäumler 2008	Osteoarthritis	Helixor M, ic, daily-1/5d	up to 20	up to 160*	30	2-4 wk	Rehabilitation	Safety: Grading of LR		LR
Bergmann 2008	Various Ca.	Aviscumine, sc, 2/wk		up to 700*	26	3-39 (6) wk	no	*Peripheral blood:* IL-1ß, IL-6, IL-10, IFN-γ, TNF-α, neutrophils<br>Safety: Every 3 wk: Toxicity (CTC), tolerability	↑ IL-1ß, IFN-γ, TNF-α;<br>↓ IL-6, IL-10	LR, fever, chills, headache, fatigue, nausea/vomiting, hypertension (further events, lab. change, see text)
Büssing 2007	Breast, colorectal, prostate Ca.	Iscador M, Qu, sc, 2-3/wk	up to 20		67	6 mth	no	*Peripheral blood*: DBC, lymphocyte subsets (CD3, CD4, CD8, CD16, CD19, CD25,CD28, CD56, CD62L, HLA-DR)<br>Safety: LR (diary)	↔ CD3, CD4, CD8, CD4/CD8<br>↓ CD25 on PHA-stimulated CD3 cells; HLA-DR CD3 cells in one subgroup	LR
Bar-Sela 2006	Ascites, various Ca.	Iscador M, ip	10		23	up to 3x		Safety: Symptom questionnaire		Abdominal pain (1 pat.), no other ADR

Author, reference	Diagnosis	Preparation, application	Dosage per application: Viscum album mg	Dosage per application: ML ng	Study size	Treatment (observation) time	Concomitant therapy	Immune parameter investigated<br>*Compartment investigated*<br>Safety: Assessing frequency and method	Immune outcome compared to pre-treatment<br>↑ increase, ↓ decrease,<br>↔ no change	Safety outcome
Schink 2006	Breast, colon Ca.	Helixor M, sc, 3/wk	1 to 100	up to 400-1000	40	up to 2 yr	no	*Peripheral blood:* NK-activity against autologous tumour and K562 cells, granulocytes, monocytes, lymphocytes, NK-, B-, T-cells, CD4, CD8<br>Safety: Regularly: Lab. parameter, AE, toxicity	↑ NK cell number<br>↔ NK cell activity	No data
Elsässer-Beile 2005	Bladder Ca.	Lektinol, intravesical, 1/wk		up to 250000*	30	6 wk		*Urine:* IL-1α, IL-2, IL-10, IFN-γ, TNF-α<br>*Plasma*: TNF-p75 receptor<br>Safety: Side effects according to WHO	↔ Cytokines, TNF-p75-receptor	No ADR, good tolerability
Matthes 2005	Colon Ca.	Abnobaviscum Fr, Qu, Helixor M; it	60, 60 or 500 resp.	42000, 25000 or 2000-5000 resp.	14	(median 8 d)	unknown	*Tumour tissue:* Peritumoural inflammation, eosinophils, B-, T-lymphocytes, CD4, CD8, macrophages/monocytes, mast cells, NK cells<br>Safety: No details	↑ B-, T-lymphocytes, CD4, macrophages, NK cells, eosinophils<br>↔ Inflammatory infiltrate, mast cells, CD8 cells	No severe ADR
Schöffski 2005	Various Ca.	Aviscumin, iv, 1/wk		up to 420000*	14	3-24 wk	no	*Peripheral blood:* DBC<br>Safety: Weekly: Toxicity, vital signs, lab. parameter; three-weekly: toxicity according to NCI CTC	No neutropenia or granulocytopenia, no haematological toxicity	Fatigue, fever, rigours/chills, urinary frequency, pruritus; reversible liver toxicity, anaphylactic reaction (further events, lab. changes, see text)
Bar-Sela 2004	Colorectal Ca.	Abnobaviscum Qu, sc, 3/wk	up to 15	up to 6000	25	4-85 wk		Safety: Monthly: toxicity according to NCI CTC incl. lab. parameters		LR, rise in temperature, eosinophilia; no other VAE-related AEs
Mabed 2004	Liver Ca.	Abnobaviscum Fr, sc, 1/wk	40	20000*	23	3-152 wk	no	*Peripheral blood:* CBC, DBC<br>Safety: Toxicity according to WHO criteria, incl. lab. parameters	No toxicity	LR, fever, pain
Schleyerbach 2004	Breast Ca.	Abnobaviscum M, sc, 2-3/wk	up to 20	up to 5800*	54	24 mth	partly CT, RT, HT	*Peripheral blood:* Eosinophils<br>Safety: No systematic assessment	↑ Eosinophils	LR, generalised urticaria

Author, reference	Diagnosis	Preparation, application	Dosage per application		Study size	Treatment (observation) time	Concomitant therapy	Immune parameter investigated<br>*Compartment investigated*<br>Safety: Assessing frequency and method	Immune outcome compared to pre-treatment<br>↑ increase, ↓ decrease,<br>↔ no change	Safety outcome
			Viscum album mg	ML ng						
Schöffski 2004	Various Ca.	Aviscumin, iv, 2/wk		up to 448000*	41	3-24 wk	no	*Peripheral blood:* DBC, IL-1ß, IL-6, IL-10, IL-12, IFN-γ, TNF-α<br>Safety: Weekly: Toxicity, vital signs, lab. parameter; three-weekly: Toxicity according to NCI CTC	↑ IL-1ß, IL-6, IFN-γ (partly)<br>4 x Mild leukopenia (<10%, grade 1); no haematological toxicity/granulocytopenia	Fatigue, fever, nausea, vomiting, allergic reaction; reversible liver toxicity (further events, lab. changes, see text)
Montes 2002	Anal condyloma	Iscador, sc, 2/wk	up to 10		51	16 wk<br>(9 mth)		Safety: No details		Rise in temperature
Huber 2001	Chronic hepatitis C	Abnobaviscum Qu, sc, 3/wk	up to 20	up to 9000	25	6 (12) mth	no	*Peripheral blood:* DBC, lymphocyte subsets<br>Safety: No details	↑ Eosinophils<br>↓ CD3, CD4 within normal range	LR, no ADR
Kang 2001	Ascites, ovarian Ca.	Helixor M, ip, 3/wk	up to 500		10	5-7 wk		Safety: No details		3 short-termed ADRs, no specification
Tusenius 2001	Chronic hepatitis C	Iscador Qu sp., sc, 3/wk	up to 10	up to 760*	5	12 (18) mth	no	*Peripheral blood:* CBC<br>Safety: Bimonthly, bi-annual: Toxicity according to WHO criteria, incl. lab. Parameters	"Not any systemic toxicity"	LR, no ADR
Kovacs 2000	Various Ca.	Iscador M, M sp.,<br>sc, 2-3/wk	up to 5	up to 250*	16	3-4 wk	no	*Peripheral blood:* IL-12; PMBC-released IFN-γ, IL-2, IL-4; CD4, CD16/CD56, CD14	↑ IL-12, IFN-γ, IL-2, monocytes (CD14)	
Büssing 1999	Various Ca.	Helixor P, M, A, sc, 2-3/wk	up to 30, 100 or 50 resp.	up to 570, 430 or 260 resp.*	23	7 mth	no	*Peripheral blood:* Leukocytes, lymphocytes, CD19, CD3, CD4, CD8, CD8/28, CD8/28-, CD16/56, CD25, HLA-DR<br>Safety: No details	↑ NK cells, relative count of lymphocytes, most parameters<br>↓ Leukocytes (non-significant)	LR
Mahfouz 1999	Breast Ca.	Abnobaviscum Fr, sc, it, 1/wk	60	30000*	26	16-136 (16-136) wk	no	Safety: Weekly: Clinical, lab. Parameters		No toxic side effects
Stein 1998	Breast Ca.	Abnobaviscum M, sc, 2/wk	0.4	200	8	16 wk	no	*Peripheral blood:* Cytokines; proliferation & cytokine release of lymphocytes	↑ Proliferation (4 wk), IFN-γ/IL-2<br>↓ TNF-α/IL-6	

Author, reference	Diagnosis	Preparation, application	Dosage per application		Study size	Treatment (observation) time	Concomitant therapy	Immune parameter investigated *Compartment investigated* Safety: Assessing frequency and method	Immune outcome compared to pre-treatment ↑ increase, ↓ decrease, ↔ no change	Safety outcome
			Viscum album mg	ML ng						
Chernyshov 1997	Children, immune-suppressed / Chernobyl	Iscador M, sc, 2/wk	0.1 - 5	1 ng/kg*	30	3 wk	no	*Peripheral blood:* Lymphocyte subsets (CD4/CD3, CD8/CD3, CD3, CD19, CD3-/CD16/CD56), PHA-stimulation of lymphocytes, NK cell activity, phagocytosis (neutrophils) Safety: Regular recording of symptoms (physician, parents)	↑ NK cell activity, lymphocyte response, phagocytosis; leukocytes (initially normal), lymphocytes (initially low); CD3, CD4/CD3, CD8/CD3, CD19, CD3-/CD16/CD56<br>↓ CD4/CD3, CD8/CD3 (initially high)	LR, anxiety during sleep (1x), muscular, joint, bone pain
Ghaleb 1997	Breast, brain Ca., pleural effusion	Abnobaviscum Fr, sc or ipl, 1/wk	60 or 100	42000 or 70000 resp.	30	16 (sc) or 6 (ipl) wk	no	*Peripheral blood:* Leukocytes, lymphocytes, granulocytes, monocytes Safety: Every 3–4 wk: Liver, kidney function tests (lab. parameters)	↑ Leukocytes (in leukopenia), lymphocytes, monocytes, eosinophils<br>↓ Leukocytes (in leukocytosis)	Cutaneous rash; no other ADR, no liver or kidney toxicity
Böcher 1996	Various Ca.	Helixor M or P, iv, 1-2/wk - 2/yr	up to 1500 (mean 600)	up to 6000-45000 (mean 2400-18000)	21	(152 ±103 d)	no	*Peripheral blood:* DBC Safety: AEs, lab. parameter, according to WHO criteria	No change	No ADR
Büssing 1996	Various Ca.	Helixor M or P or A, iv, once	up to 800	up to 8000-24000	12	(1 wk)		*Peripheral blood:* DBC, lymphocyte subsets, CRP Safety: No details	↑ Juvenile granulocytes, monocytes, CD4/CD8, CD25CD3	No inflammatory ADR
Kuehn 1996	Breast Ca.	Iscador M, sc, twice	up to 2	up to 100	12	1 wk	no	*Peripheral blood:* Leukocytes, lymphocytes, monocytes, granulocytes, NK cell-activity, CD56/CD16, CD3, CD19	↑ Leukocytes, granulocytes<br>↓ Lymphocytes, CD3, (normal range)<br>↔ B-cells	
Kuehn 1996	Breast Ca.	Iscador M and M sp, sc, 2/wk	up to 5	up to 250	42	3-4 wk	no	*Peripheral blood:* Leukocytes, lymphocytes, monocytes, granulocytes, NK cell-activity, CD56/CD16, CD3, CD19	↑ Leukocytes, granulocytes, NK-cells	
Henn 1995	Breast Ca.	Abnobaviscum M, sc, 2/wk	0.3 – 0.76	up to 380*	10	3 (16) wk	no	*Peripheral blood:* DBC, LGL Safety: LR	↑ Leukocytes, granulocytes, eosinophils, monocytes, LGL, lymphocytes	LR

Author, reference	Diagnosis	Preparation, application	Dosage per application		Study size	Treatment (observation) time	Concomitant therapy	Immune parameter investigated *Compartment investigated* Safety: Assessing frequency and method	Immune outcome compared to pre-treatment ↑ increase, ↓ decrease, ↔ no change	Safety outcome
			Viscum album mg	ML ng						
Lukyanova 1994	Children, immune-suppressed/ Chernobyl	Iscador M, sc, 2/wk	up to 5	up to 32.5*	25	5 wk (15 mth)	no	*Peripheral blood:* Lymphocyte subsets, phagocytosis, IgG, IgA, IgM, C3, C4 *Saliva*: Secretory IgA *Clinical:* Respiratory infections Safety: Regular recording of symptoms (physician), physical examination	↓ Incidence of respiratory infections<br>↑ CD3, CD19, CD16, phagocytosis, C4, IgM, IgA, sIgA<br>↔ CD4, CD8, C3, IgG	LR; no other ADR
Stumpf 1994	Malignant pleural effusion	Helixor A, M, P, ipl	up to 1000, 600 or 200 resp.	up to 7000 or 6000 or 6000 resp.	18	1-12 mth	Partly CT, HT	*Pleural effusion:* Lymphocyte subsets, anti-ML-AB Safety: No details	Anti-ML-AB, but not lymphocytes correlate to VAE-instillations.	Pain, burning (grade 1)
Salzer 1990, Böck 1980	Malignant pleural effusion	Iscador M, P, Qu, ipl	50		9			*Peripheral blood, pleural effusion:* OKT3, OKB7, OKT4, OKT8, OKNK, OKDR, OKT4/DR, OKT4/OKT8 Safety: No details	↑ OKT4/OKT8, eosinophils, lymphocytes<br>↓ OKT8 (pleural effusion)	Fever, chills, moderate pleurodynia
Hajto 1989	Breast Ca.	Iscador MSF, iv, QuFrF, sc, once	23 or 12 resp.	115 or 840 resp.*	31	(48 h)	no	*Peripheral blood:* Segmented and young neutrophils, lymphocytes, phagocytosis (granulocytes), LGL	↑ All parameters (transient decrease of LGL)	
Hajto 1986	Breast Ca.	Iscador M 2%, iv, once	14.7 – 26.6		20	(72 h)	no	*Peripheral blood:* Phagocytosis (granulocytes), NK-, ADCC-activity, neutrophils, lymphocytes, LGL, LTT, others Safety: No details	↑ All parameters (transient decrease of lymphocytes, LGL, NK-, ADCC-activity)	Fever, headache, nausea, chills

Abbreviations (see also text): AB: antibody, ADCC: antibody-dependent-cell-mediated cytotoxicity, AE: Adverse event, AZT: azidothymidine, BCG: Bacille Calmette-Guérin, C3 or 4: complement component 3 or 4, CAF: Cyclophosphamide, Adriamycin, 5-Fluorouracil, CBC complete blood count, CMF: Cyclophosphamide, Methotrexate, 5-Fluorouracil, CRP: C-reactive protein, CT: chemotherapy, DBC: differential blood count, DDI: Didanosine, DDC: dideoxycytidine, ECG: electrocardiogram, ENT: ear, nose, throat, 5-FU: 5-Fluorouracil, G(M)-CSF: granulocyte (macrophage) colony stimulating factor, HB haemoglobin, HT: hormone therapy, IFNγ: interferon γ, Ig: immunoglobuline, IP: Iscador Pini, IP10: interferon gamma-induced protein 10 kDa, ip: intraperitoneal, ipl: intrapleural, IQ: Iscador Querci, it: intratumoural, iv: intravenous, mth: month(s), lab.: laboratory, LGL: large granular lymphocyte, LTT: lymphocyte transformation test, NCI CTC: National Cancer Institute – Common Toxicity Criteria, PHA: phytohaemagglutinin PMA: phorbol myristate acetate, PMBC: peripheral blood mononuclear cell, RT: radiotherapy, resp.: respectively, sAE: serious adverse event; sc: subcutaneous, sp.: special, TGFß: transforming (tumour) growth factor ß, TNFα/ ß: tumour necrosis factor α/ß, wk: week(s), yr: year(s).

Reference List

Auerbach L, Dostal V, Václavik-Fleck I, Kubista E, Rosenberger A, Rieger S, Tröger W, Schierholz JM: **Signifikant höherer Anteil aktivierter NK-Zellen durch additive Misteltherapie bei chemotherapierten Mamma-Ca-Patientinnen in einer prospektiven randomisierten doppelblinden Studie.** In: *Fortschritte in der Misteltherapie. Aktueller Stand der Forschung und klinischen Anwendung*. Edited by Scheer R, Bauer R, Becker H, Fintelmann V, Kemper FH, Schilcher H. Essen, KVC Verlag 2005, 543-554

Bar-Sela G, Goldberg H, Beck D, Amit A, Kuten A: **Reducing malignant ascites accumulation by repeated intraperitoneal administrations of a *Viscum album* extract.** *Anticancer Res* 2006, **26:**709-714

Bar-Sela G, Haim N: **Abnobaviscum (mistletoe extract) in metastatic colorectal carcinoma resistant to 5-fluorouracil and leucovorin-based chemotherapy.** *Med Oncol* 2004, **21:**251-254

Bäumler S: **Die Wirkung der Mistel bei degenerativen Erkrankungen des Bewegungsapparates-eine prospektive Falldokumentation.** *Komplement Integr Med* 2008, **49:**31-37

Bergmann L, Aamdal S, Marreaud S, Lacombe D, Herold M, Yamaguchi T, Wilhelm-Ogunbiyi K, Lentzen H, Zwierzina H: **Phase I trial of r viscumin (INN: aviscumine) given subcutaneously in patients with advanced cancer: A study of the European Organisation for Research and Treatment of Cancer (EORTC protocol number 13001).** *Eur J Cancer* 2008, **44:**1657-1662

Böcher E, Stumpf C, Büssing A, Schietzel M: **Prospektive Bewertung der Toxizität hochdosierter *Viscum album* L.-Infusionen bei Patienten mit progredienten Malignomen.** *Z Onkol* 1996, **28:**97-106

Böck D, Salzer G: **Morphologischer Nachweis einer Wirksamkeit der Iscadorbehandlung maligner Pleuraergüsse und ihre klinischen Ergebnisse.** *Krebsgeschehen* 1980, **12:**49-53

Büssing A, Brückner U, Enser-Weis U, Schnelle M, Schumann A, Schietzel M, Hatzmann W, Hackmann J: **Modulation of chemotherapy-associated immunosuppression by intravenous application of *Viscum album* L. extract (Iscador): a randomised phase II study.** *Eur J Integr Med* 2008, **1:**S44-S54

Büssing A, Rosenberger A, Stumpf C, Schietzel M: **Entwicklung lymphozytärer Subpopulationen bei Tumorpatienten nach subkutaner Applikation von Mistelextrakten.** *Forsch Komplementmed* 1999, **6:**196-204

Büssing A, Stumpf C, Stumpf RT, Wutte H, Schietzel M: **Therapiebegleitende Untersuchung immunologischer Parameter bei Tumor-Patienten nach hochdosierter intravenöser Applikation von *Viscum album* L.-Extrakten.** *Z Onkol* 1996, **28:**54-59

Büssing A, Stumpf C, Tröger W, Schietzel M: **Course of mitogen-stimulated T lymphocytes in cancer patients treated with *Viscum album* extracts.** *Anticancer Res* 2007, **27:**2903-2910

Cazacu M, Domsa I, Stugren C, Oniu T, Muresan F: **Inflammatory reaction in colo-rectal cancer patients treated with *Viscum album* total extract.** *Radioterapie & Oncologie Medicala* 2006, **1:**54-58

Cazacu M, Oniu T, Lungoci C, Mihailov A, Cipak A, Klinger R, Weiss T, Zarkovic N: **The influence of Isorel on the advanced colorectal cancer.** *Cancer Biother Radiopharm* 2003, **18:**27-34

Chernyshov VP, Omelchenko LI, Heusser P, Slukvin II, Vodyanik MA, Galazyuk LV, Vykhovanets EV, Pochinok TV, Chernyshov AV, Gumenyuk ME, Schaefermeyer H, Schaefermeyer G: **Immunomodulatory actions of *Viscum album* (Iscador) in children with recurrent respiratory disease as a result of the Chernobyl nuclear accident.** *Complement Ther Med* 1997, 141-146

Dold U, Edler L, Mäurer HCh, Müller-Wening D, Sakellariou B, Trendelenburg F, Wagner G: *Krebszusatztherapie beim fortgeschrittenen nicht-kleinzelligen Bronchialkarzinom.* Stuttgart, New York: Georg Thieme Verlag 1991

Douwes FR, Kalden M, Frank G, Holzhauer P: **Behandlung des fortgeschrittenen kolorektalen Karzinoms.** *Dtsch Zschr Onkol* 1988, **20:**63-67

Douwes FR, Wolfrum DI, Migeod F: **Ergebnisse einer prospektiv randomisierten Studie: Chemotherapie versus Chemotherapie plus "Biological Response Modifier" bei metastasierendem kolorektalen Karzinom.** *Krebsgeschehen* 1986, **18:**155-163

Ebrahim MA, El-Hadaad HA, Alemam OA, Keshta SA: **Efficacy and safety of viscum fraxini-2 in advanced hepatocellular carcinoma: a phase II study.** *Chin Ger J Clin Oncol* 2010, **9:**452-458

Eisenbraun J, Scheer R, Kröz M, Schad F, Huber R: **Quality of life in breast cancer patients during chemotherapy and concurrent therapy with a mistletoe extract.** *Phytomedicine* 2011, **18:**151-157

Elsässer-Beile U, Leiber C, Wolf P, Lucht M, Mengs U, Wetterauer U: **Adjuvant intravesical treatment of superficial bladder cancer with a standardized mistletoe extract.** *The Journal of Urology* 2005, **174:**76-79

Enesel MB, Acalovschi I, Grosu V, Sbarcea A, Rusu C, Dobre A, Weiss T, Zarkovic K: **Perioperative application of the *Viscum album* extract Isorel in digestive tract cancer patients.** *Anticancer Res* 2005, **25:**4583-4590

Fellmer Ch, Fellmer KE: **Nachbehandlung bestrahlter Genitalkarzinome mit dem Viscum-album-Präparat "Iscador".** *Krebsarzt* 1966, **21:**174-185

Ghaleb HA. **Report on Abnoba viscum - Multicentral clinical trial.** 1997.

Gorter RW, Stein J, Stoss M, Linder M: **Prospektive, longitudinale, Dosis-eskalierende, randomisierte Phase-I/II-Studie mit Iscador QuFrF und Iscador Qu Spezial mit HIV-Positiven, Krebspatienten und gesunden, nichtrauchenden Probanden.** *Forsch Komplementmed* 1996, **3:**169-175

Gorter RW, van Wely M, Reif M, Stoss M: **Tolerability of an extract of European mistletoe among immunocompromised and healthy individuals.** *Altern Ther Health Med* 1999, **5:**37-48

Gorter RW, van Wely M, Stoss M, Wollina U: **Subcutaneous infiltrates induced by injection of mistletoe extracts (Iscador).** *Am J Ther* 1998, **5:**181-187

Gutsch J, Berger H, Scholz G, Denck H: **Prospektive Studie beim radikal operierten Mammakarzinom mit Polychemotherapie, Helixor und unbehandelter Kontrolle.** *Dtsch Zschr Onkol* 1988, 94-100

Hajto T: **Immunomodulatory effects of Iscador: a *Viscum album* preparation.** *Oncology* 1986, **43:**51-65

Hajto T, Hostanska K: **An investigation of the ability of *Viscum album*-activated granulocytes to regulate natural killer cells in vivo.** *Clin Trials J* 1986, **23:**345-358

Hajto T, Hostanska K, Gabius H-J: **Modulatory potency of the β-galactoside-specific lectin from mistletoe extract (Iscador) on the host defense system in vivo in Rabbits and patients.** *Cancer Res* 1989, 4803-4808

Hajto T, Lanzrein C: **Natural killer and antibody-dependent cell-mediated cytotoxicity activities and large granular lymphocyte frequencies in *Viscum album*-treated breast cancer patients.** *Oncology* 1986, **43:**93-97

Hekal IA, Samer T, Ibrahim EI: **Viscum Fraxini 2, as an adjuvant therapy after resection of superficial bladder cancer: Prospective clinical randomized study. Presented at the 43rd Annual Congress of The Egyptian Urological Association in conjunction with The European Association of Urology November 10-14, 2008 Hurghada, Egypt. Abstract P8.** 120.

Henn W: **Verlauf der Körperkerntemperatur und der Zahl peripherer Blutzellen unter Berücksichtigung ihrer Zirkadianrhythmik bei Mammakarzinom-Patientinnen vor und während der Therapie mit einem Mistelpräparat - Eine explorative Studie.** *PhD Thesis.* 1995.

Huber R, Classen K, Werner M, Klein R: **In vitro immunoreactivity towards lectin-rich or viscotoxin-rich mistletoe (Viscum album L.) extracts Iscador applied to healthy individuals.** *Arzneimittelforschung* 2006, **56:**447-456

Huber R, Eisenbraun J, Miletzki B, Adler M, Scheer R, Klein R, Gleiter CH: **Pharmacokinetics of natural mistletoe lectins after subcutaneous injection.** *Eur J Clin Pharmacol* 2010, DOI 10.1007/s00228-010-0830-5

Huber R, Ellwanger U, Wieber J, Beckmann C: **Safety and immunological effects of Iscucin« Populi and Viscum Mali--A placebo-controlled study.** *Eur J Integr Med* 2009, **1:**183

Huber R, Klein R, Berg PA, Lüdtke R, Werner M: **Effects of a lectin- and a viscotoxin-rich mistletoe preparation on clinical and hematologic parameters: a placebo-controlled evaluation in healthy subjects.** *J Altern Complement Med* 2002, **8:**857-866

Huber R, Klein R, Lüdtke R, Werner M: **Häufigkeit grippaler Infekte bei Gesunden unter Gabe eines lektinreichen und eines lektinarmen Mistelpräparats im Rahmen einer randomisierten, doppelblinden, placebokontrollierten Studie.** *Forsch Komplementmed* 2001, **8:**354-358

Huber R, Lüdtke R, Klassen M, Muller-Buscher G, Wolff-Vorbeck G, Scheer R: **Effects of a mistletoe preparation with defined lectin content on chronic hepatitis C: an individually controlled cohort study.** *Eur J Med Res* 2001, **6:**399-405

Huber R, Rostock M, Goedl R, Ludtke R, Urech K, Buck S, Klein R: **Mistletoe treatment induces GM-CSF- and IL-5 production by PBMC and increases blood granulocyte- and eosinophil counts: a placebo controlled randomized study in healthy subjects.** *Eur J Med Res* 2005, **10:**411-418

Huber R, Rostock M, Goedl R, Ludtke R, Urech K, Klein R: **Immunologic effects of mistletoe lectins: a placebo-controlled study in healthy subjects.** *J Soc Integr Oncol* 2006, **4:**3-7

Kang SB. **Efficacy of Mistletoe in the Management of Malignant Ascites.** 2001. 35.Medizinische Woche Baden-Baden, 27-28 Oct.

Klein R, Claßen K, Berg PA, Lüdtke R, Werner M, Huber R: **In vivo-induction of antibodies to mistletoe lectin-1 and viscotoxin by exposure to aqueous mistletoe extracts: A randomised double-blinded placebo controlled phase I study in healthy individuals.** *Eur J Med Res* 2002, **7:**155-163

Klose C, Jensen K, Herzig M, Mansmann U: *Multicentric, randomized, open, prospective clinical trial for the investigation of efficacy and tolerance and adverse drug reactions of HELIXOR® A in comparison to Lentinan in patients with non small cell lung cancer, breast cancer or ovarian cancer. Forschungsberichte der Abteilung Medizinische Biometrie, Universität Heidelberg. 2003.* 2003.

Kovacs E: **Serum levels of IL-12 and the production of IFN-gamma, IL-2 and IL-4 by peripheral blood mononuclear cells (PBMC) in cancer patients treated with *Viscum album* extract.** *Biomed Pharmacother* 2000, **54:**305-310

Kuehn JJ, Fornalski M: **Beeinflussung immunkompetenter Zellen des peripheren Blutes durch *Viscum album* (Iscador M) bei Patientinnen mit Mammakarzinom.** In: *Grundlagen der Misteltherapie. Aktueller Stand der Forschung und klinische Anwendung*. Edited by Scheer R, Becker H, Berg PA. Stuttgart, Hippokrates Verlag GmbH 1996, 366-379

Lange O, Scholz G, Gutsch J. **Modulation der subjektiven und objektiven Toxizität einer aggressiven Chemotherapie mit Helixor. Unpublished Report.** 1985.

Loewe-Mesch A, Kuehn JH, Borho K, Abel U, Bauer C, Gerhard I, Schneeweiss A, Sohn C, Strowitzki T, Hagens C: **Adjuvante simultane Mistel-/Chemotherapie bei Mammakarzinom – Einfluss auf Immunparameter, Lebensqualität und Verträglichkeit.** *Forsch Komplementmed* 2008, **15:**22-30

Longhi A, Mariani E, Kuehn JJ: **A randomized study with adjuvant mistletoe versus oral Etoposide on post relapse disease-free survival in osteosarcoma patients.** *Eur J Integr Med* 2009, **1:**27-33

Lukyanova EM, Chernyshov VP, Omelchenko LI, Slukvin II, Pochinok TV, Antipkin JG, Voichenko IV, Heusser P, Schniedermann G: **Die Behandlung immunsupprimierter Kinder nach dem Tschernobyl-Unfall mit *Viscum album* (Iscador): Klinische und immunologische Untersuchungen.** *Forsch Komplementmed* 1994, **1:**58-70

Mabed M, El-Helw L, Sharma S: **Phase II study of viscum fraxini-2 in patients with advanced hepatocellular carcinoma.** *Br J Cancer* 2004, **90:**65-69

Mahfouz MM, Ghaleb HA, Hamza MR, Fares L, Moussa L, Moustafua A, El-Za Wawy A, Kourashy L, Mobarak L, Saed S, Fouad F, Tony O, Tohamy A: **Multicenter open labeled clinical study in advanced breast cancer patients. A preliminary report.** *Journal of the Egyptian Nat Cancer Inst* 1999, **11:**221-227

Mansky PJ, Wallerstedt DB, Monahan BP, Lee C, Sannes T, Stagl J, Blackman MA, Swain SL, Grem J: **Phase I study of mistletoe extract/gemcitabine combination treatment in patients with advanced solid tumors.** *Onkologie* 2008, **31:**200

Mansky PJ, Wallerstedt DB, Sannes T, Stagl J, Johnson L, Blackmann MR, Grem J, Swain SM, Monahan BP: **NCCAM/NCI phase I study of mistletoe extract and gemcitabine in patients with advanced solid tumors.** *J Clin Oncol* 2010, **28:**abstr 2559

Matthes B, Fritz P, Mürdter TE, Kröz M, von Laue H-B, Matthes H: **Untersuchungen zu Immunreaktion und klinischem Outcome nach intraläsionaler *Viscum*-Applikation bei Kolonkarzinom.** In: *Fortschritte in der Misteltherapie. Aktueller Stand der Forschung und klinischen Anwendung*. Edited by Scheer R, Bauer R, Becker H, Fintelmann V, Kemper FH, Schilcher H. Essen, KVC Verlag 2005, 491-498

Montes JR, Picconi MA, Cabanne AM, Muñoz P, Masciangiolo G, Barrera A, Alonio LV, Teyssié AR: **HPV anogenital lesions treated with Viscum album. Preliminary report. Conferencia Mundial de HPV.**

Musielski H, Rüger K: **Verfahren zur quantitativen Bestimmung von Mistellektin I und Mistellektin II und/oder Mistellektin III in Mistelextrakten unter Verwendung monoklonaler Antikörper, die spezifisch mit Mistellektin reagieren.** In: *Grundlagen der Misteltherapie. Aktueller Stand der Forschung und klinische Anwendung*. Edited by Scheer R, Becker H, Berg PA. Stuttgart, Hippokrates Verlag GmbH 1996, 95-104

Piao BK, Wang YX, Xie GR, Mansmann U, Matthes H, Beuth J, Lin HS: **Impact of complementary mistletoe extract treatment on quality of life in breast, ovarian and non-small cell lung cancer patients. A prospective randomized controlled clinical trial.** *Anticancer Res* 2004, **24:**303-309

Salzer G, Popp W: **Die lokale Iscadorbehandlung der Pleurakarzinose.** In: *Krebs und Alternativmedizin II*. *Volume II.* Edited by Jungi WF, Senn H-J. Berlin - Heidelberg, Springer-Verlag 1990, 36-49

Schink M, Tröger W, Dabidian A, Goyert A, Scheuerecker H, Meyer J, Fischer IU, Glaser F: **Mistletoe extract reduces the surgical suppression of natural killer cell activity in cancer patients. A randomized phase III trial.** *Forsch Komplementmed* 2007, **14:**9-17

Schink M, Tröger W, Goyert A, Scheuerecker H, Selbmann K, Glaser F: **Zusammenhang der NK-Zellaktivität gegen autologe Tumor- und K562-Zellen mit dem klinischen Verlauf unter Misteltherapie.** *Forsch Komplementmed* 2006, **13:**147-155

Schleyerbach PW: **Verlauf immunologischer Parameter bei Mammakarzinom unter Therapie mit Mistel-Extrakten: eine Anwendungsbeobachtung.** *PhD Thesis.* Eberhard-Karls-Universität zu Tübingen; 2004.

Schöffski P, Breidenbach I, Krauter J, Bolte O, Stadler M, Ganser A, Wilhelm-Ogunbiyi K, Lentzen H: **Weekly 24 h infusion of aviscumine (rViscumin): a phase I study in patients with solid tumours.** *Eur J Cancer* 2005, **41:**1431-1438

Schöffski P, Riggert S, Fumoleau P, Campone M, Bolte O, Marreaud S, Lacombe D, Baron B, Herold M, Zwierzina H, Wilhelm-Ogunbiyi K, Lentzen H, Twelves C, European Organization for Research and Treatment of Cancer New Drug Development Group: **Phase I trial on intravenous aviscumine (rViscumin) in patients with solid tumors: a study of the European Organization for Research and Treatment of Cancer New Drug Development Group.** *Ann Oncol* 2004, **15:**1816-1824

Son GS, Ryu WS, Kim HY, Woo SU, Park KH, Bae JW: **Immunologic Response to Mistletoe Extract (Viscum album L.) after Conventional Treatment in Patients with Operable Breast Cancer.** *J Breast Cancer* 2010, **13:**14-18

Stein GM, Berg PA: **Modulation of cellular and humoral immune responses during exposure of healthy individuals to an aqueous mistletoe extract.** *Eur J Med Res* 1998, **3:**307-314

Stein GM, Henn W, von Laue HB, Berg PA: **Modulation of the cellular and humoral immune responses of tumor patients by mistletoe therapy.** *Eur J Med Res* 1998, **3:**194-202

Stoss M, Gorter RW: **No evidence of IFN-γ increase in the serum of HIV-positive and healthy subjects after subcutaneous injection of a non-fermented *Viscum album* L. extract.** *Nat Immun* 1998, **16:**157-164

Stoss M, van Wely M, Musielski H, Gorter RW: **Study on local inflammatory reactions and other parameters during subcutaneous mistletoe application in HIV-positive patients and HIV-negative subjects over a period of 18 weeks.** *ArzneimForsch/DrugRes* 1999, **49(I):**366-373

Stumpf C, Schietzel M: **Intrapleurale Instillation eines Extraktes aus *Viscum album* [L.] zur Behandlung maligner Pleuraergüsse.** *Tumordiagnose und Therapie* 1994, 57-62

Tröger W, Jezdic S, Ždrale Z, Tisma N, Hamre HJ, Matijasevic M: **Quality of Life and Neutropenia in Patients with Early Stage Breast Cancer: A Randomized Pilot Study Comparing Additional Treatment with Mistletoe Extract to Chemotherapy Alone.** *Breast Cancer* 2009, **3:**35-45

Tröger W, Matijasevic M: *Study report: Additional therapy with Helixor and Iscador in breast cancer patients receiving chemotherapy: A prospective randomized open-label study.* 2008.

Tusenius KJ, Spoek JM, Kramers CW: **Iscador Qu for chronic hepatitis C: an exploratory study.** *Complement Ther Med* 2001, **9:**12-16

van Wely M, Stoss M, Gorter RW: **Toxicity of a standardized mistletoe extract in immunocomprised and healthy individuals.** *Am J Ther* 1999, **6:**37-43

Wispler M: **Prospektive, randomisierte, monozentrische Studie zur Aktivierung des Homings und des allgemeinen Aktivitätsniveaus von T-Lymphozyten durch subkutane Mistel-Extrakt-Injektionen bei gesunden männlichen Probanden. Inaugural-Dissertation. Universität Witten/Herdecke.** *PhD Thesis.* 2001.

Wispler M, Kappler M, Soto-Vera D, Reif M, Schnelle M, Zänker KS: **Prospektive, randomisierte, kontrollierte Studie zur Aktivierung des Homings und des allgemeinen Aktivitätsniveaus von T-Lymphozyten durch subkutane Mistelextrakt-Injektionen bei gesunden männlichen Probanden.** In: *Fortschritte in der Misteltherapie. Aktueller Stand der Forschung und klinischen Anwendung*. Edited by Scheer R, Bauer R, Becker H, Fintelmann V, Kemper FH, Schilcher H. Essen, KVC Verlag 2005, 513-526
