# Supplementary material for: Safety of higher dosages of Viscum album L. in animals and humans - systematic review of immune changes and safety parameters
Source: BMC Complement Altern Med. 2011 Aug 28;11:72. doi: 10.1186/1472-6882-11-72 (PMC3180269; doi:10.1186/1472-6882-11-72)
Supplement: Additional file 3 — Animal experiments investigating Viscum album > 0.02 mg/kg or ML > 1 ng/kg body weight and comparing immune and safety outcomes with a control group. Characteristics for each animal experiment included in the review: animals, diagnosis, preparation, application, dosage per application, treatment (follow-up) period, concomitant therapy, immune and safety parameter investigated, compartment investigated, assessing frequency (safety parameter), immune outcomes compared to control group, safety outcome, and citation. [file 1472-6882-11-72-S3.PDF]

Additional file 3 – Animal experiments investigating Viscum album > 0.02 mg/kg or ML > 1 ng/kg body weight and comparing immune and safety outcomes with a control group (concerning ML contents see also additional file 2).

Author, reference	Animals, diagnosis	Preparation, application	Dosage per application		Treatment (follow-up) period	Conco-mitant therapy	Immune Parameter Investigated<br>*Compartment investigated*<br>Safety: Assessing frequency and method	Immune outcome compared to control group<br>↑ above, ↓ below,<br>↔ no difference	Safety outcome
			Viscum album mg/kg	ML ng/kg					
**Healthy animals**									
Gren 2009	Mice	Iscador Qu or M or P, ip, once	5	400, 260 or 20 resp.	(24 h)	no	*Peripheral blood:* Relative amount (%) of lymphocytes, monocytes, neutrophils; total protein, albumin, globulin	↑ % of lymphocytes; albumin<br>↓ % of neutrophils, % of monocytes; total protein, globulin (α2, ß, γ)	
Hajto 2006	Mice	Iscador M sp., sc, once or 2/wk resp.	2.1, 0.42 or 0.08	112.5, 22.5 or 4.5*	(1) or 28 (29) d resp.	partly DX	*Peripheral blood:* CD4/CD8 lymphocytes<br>*Thymus:* CD4, CD8 single +, double + (DP), double – (DN) thymocytes	↑ CD4/CD8 (at low dose, single application); DN thymocytes (at 0.08 and 0.42 ng/kg)<br>↔ Parameters at 2.1 ng/kg<br>↓ DX-induced decrease of CD4/CD8, thymocytes	
Lavelle 2004	Mice	ML I, II, III oral, 1/2 wks		500000	49 (55) d	no	*Immune response:* Influence on humoral response (serum IgG, mucosal IgA) to OVA	↑ OVA-specific serum IgG, mucosal IgA	
Hajto 2003	Mice	ML I, sc, once or 2/wk resp.		1 or 30	(1), 21 (24) d resp.	partly DX	*Peripheral blood:* CD4/CD8 lymphocytes<br>*Thymus*: CD4, CD8 single + (SP), double + (DP), double – (DN) thymocytes	↑ CD4/CD8; DP, DN, CD4+, CD8+, total thymocytes (primarily at 30 ng/kg)<br>↓ DX-induced decrease of thymocytes	
Lavelle 2002	Mice	ML I, II, III, intranasal, 1/2 wks		50000	42 (56) d	no	*Immune response:* Influence on humoral (IgA, IgG) and cellular (T-cells) response to herpes simplex virus glycoprotein (gD2)	↑ gD2-specific serum IgG, mucosal IgA, T-cell proliferation	
Lavelle 2001	Mice	ML I, intranasal, 1/2 wks		50000	6 (8) wk	no	*Immune response:* Influence on humoral response (IgG, IgA) to OVA	↑ OVA-specific systemic serum IgG, mucosal IgA	

Author, reference	Animals, diagnosis	Preparation, application	Dosage per application		Treatment (follow-up) period	Conco-mitant therapy	Immune Parameter Investigated<br>*Compartment investigated*<br>Safety: Assessing frequency and method	Immune outcome compared to control group<br>↑ above, ↓ below,<br>↔ no difference	Safety outcome
			Viscum album mg/kg	ML ng/kg					
Hajto 1998	Rats	rML I, iv, once		up to 30	(48 h)	no	*Peripheral blood:* Leukocytes, lymphocytes, CD4, CD8, granulocytes, LGL, Mac-1 mononuclear cells<br>*Spleen cells:* NKR-P1A^{dim}/ NKR-P1A^{bright}; NK-activity	↑ Lymphocytes[§], Mac-1, NKR-P1A^{dim}/ NKR-P1A^{bright} (low dose)<br>↔ Leukocytes, granulocytes, CD4, CD8<br>↓ NK-activity, LGL (high dose)	
Beuth 1994	Mice	ML I sc, 4/wk		0.5 or 1 or 2.5 or 5	6 (8) d	no	*Thymus:* Size, thymocytes, L3T4, Lyt-2, L3T4/Lyt-2<br>*Peritoneal macrophages*<br>*Peripheral blood*: Leukocytes, lymphocytes, monocytes, activation markers, granulocytes<br>Safety: No details	↑ Thymocytes, Lyt-2, L3T4/Lyt-2, macrophages, leukocytes, lymphocytes, monocytes, IL-2/PBL, MAC-3/PBM (low dosages)<br>↔ Other parameter or dosages	No ADRs
Kuttan 1993	Mice	Iscador M, ip, daily	83		14 (17) d	Endoxan	*Peripheral blood:* DBC	↓ Endoxan-induced leukocytopenia	
Kuttan 1993	Mice	Iscador M ip, q2d	83		10 (20) d	RT	*Peripheral blood:* DBC	↓ Radiation-induced leukocytopenia	
Kuttan 1992	Mice	Iscador ip, once	83		(4 d)	no	*Spleen:* Mitogen-induced proliferation of lymphocytes<br>*Peritoneal macrophages*: Cytotoxicity	↑ Mitogen response; macrophage mediated cytotoxicity	
Kuttan 1992	Mice	Iscador, ip, once	83		(6 d)	no	*Immune response:* Influence on humoral response (PFC) to SRBC	↑ PFC	
Kuttan 1992	Mice	Iscador, ip, q2d	83		10 (15) d	no	*Immune response:* Influence on humoral response (circulating antibodies) to SRBC	↑ Circulating antibodies	
Joshi 1991	Mice	ML I ip, q2d		10	9 (12) d	no	*Spleen:* Size, mitogen response (DNA-synthesis) of lymphocytes, NK-cells, -activity, T-cells, B-cells<br>*Thymus*: Size	↑ Mitogen response, NK-cells, -activity, spleen size, thymus size<br>↔ T-, B-cells	
Rentea 1981	Mice	Iscador M ip, daily or 4/wk	72		2-16 wk	no	*Thymus*: Size, histologic analysis<br>*Spleen and thoracic lymph nodes*: Morphometric analysis[§]	↑ Thymus: size; hyperplasia of thymus cortex; spleen, lymph nodes: proliferation of lymphoid cells	
Rentea 1981	Mice	Iscador M ip, daily	75		6 d	no	*Thymus*: Size[§]	↑ Thymus	

Author, reference	Animals, diagnosis	Preparation, application	Dosage per application: Viscum album mg/kg	Dosage per application: ML ng/kg	Treatment (follow-up) period	Conco-mitant therapy	Immune Parameter Investigated<br>*Compartment investigated*<br>Safety: Assessing frequency and method	Immune outcome compared to control group<br>↑ above, ↓ below,<br>↔ no difference	Safety outcome
Rentea 1981	Rats	Iscador M ip, daily	19 or 38		16 d or 8 wk resp.	no	*Thymus*: Size, ConA-induced proliferation of lymphocytes	↑ Thymus, proliferation of lymphocytes	
Rentea 1981	Mice	Iscador M, ip, once or daily	14		day -3 or 4-10 (10 d)	no	*Immune response:* Influence on humoral response (antibody) to SRBC	↑ Antibody titer (treatment day 4-10)<br>↓ Antibody titer (pre-treatment: once on day 3)	
Bloksma 1979	Mice	Iscador M, ic, once	10-400		day 0 or 5 (5 d)	no	*Immune response*: Influence on cellular response (DTH) to SRBC	↑ DTH to SRBC	
Bloksma 1979	Mice	Iscador M, ip, once	20 or 80		(up to 9 d)	no	*Immune response:* Influence on humoral response (PFC) to SRBC	↑ PFC	
Stettler 1978	Mice	Iscador M ip, daily	30 – 60		7 (8) d	no	*Thymus, spleen, adjacent parathymic lymph nodes*: Weight, thymocyte count	↑ Thymus, spleen (both slight), parathymic lymph nodes; thymocytes (slight)	
**Animals partly healthy, partly with cancer**									
Jurin 1997, 96	Mice Sarcoma	Isorel M, ip once or 5/wk	14, 140, 1400		(4 d) and 2-5 wk resp.	No	*Immune response:* Influence on humoral response (PFC) to SRBC	↑ PFC at 1x and 10x application<br>↓ PFC at 25x application	
Kuttan 1992	Mice Ehrlich Ca.	Iscador, ip, once	83		(20 d)	No	*Spleen cells:* NK-activity, ADCC)	↑ NK-activity, ADCC	
**Animals with cancer – experiments**									
Seifert 2008	SCID mice Leukaemia	Helixor A or P, ip, 4-5/wk	1, 50, 100 or 1, 50 resp.	up to 320 and 725 resp.*	18 (10) d	No	*Peripheral blood*: CBC, leukocytes<br>Safety: 2/wk: careful monitoring for toxicity, body weight	↑ Leukocytes (highest dose)	Weight loss at 50 mg/kg Helixor P; no toxicity, good tolerability of Helixor A
Thies 2008	SCID mice Melanoma	ML-I, ip, daily		30, 150, 500	19 (20) d	No	*Tumour tissue:* Tumour-infiltrating dendritic cells (DCs)<br>Safety: Daily: vitality score; necropsy	↑ DCs in primary melanoma<br>↔ DCs in lung metastases	No negative effect on vitality; no VAE-related lesions
Beuth 2006	Mice Breast Ca.	Helixor A or M, it, 3/wk	250	1788 or 2615 resp.*	14 (16) d	No	*Tumour tissue:* Leukocytes, CD3, CD4, CD8, CD45<br>Safety: No details	↔ All parameters	No ADRs
Van Huyen 2006	Mice Melanoma	Iscador QuFrF, ip, daily	1	200*	7 d	No	*Spleen:* Splenocytes, CD3, CD20, CD4, CD8, mitogen-stimulated proliferation, secretion of IL-4, IL-10, IFNγ, IL-12<br>Safety: No details	↑ Mitogen-stimulated proliferation, IL-12 secretion<br>↔ Other parameters	>20 µg VAE/mouse associated with lethality

Author, reference	Animals, diagnosis	Preparation, application	Dosage per application		Treatment (follow-up) period	Conco-mitant therapy	Immune Parameter Investigated<br>*Compartment investigated*<br>Safety: Assessing frequency and method	Immune outcome compared to control group<br>↑ above, ↓ below,<br>↔ no difference		Safety outcome
			Viscum album mg/kg	ML ng/kg						
Rostock 2005	Nude mice Pancreas Ca.	Abnobaviscum Fr or MLI, it, 2/wk	4, 8 or 16	up to 10600* or 5300	28 d	No	Safety: Body weight, lethality			At 16 mg/kg body weight loss, lethality
Braun 2002	Mice Sarcoma	Iscador sp., sc, 3/wk	up to 25	> 1000	14 d	No	*Peripheral blood:* Leukocytes, lymphocytes, monocytes, granulocytes<br>*Thymus:* Thymocytes	↑<br>↔	Leukocytes, lymphocytes, monocytes, thymocytes<br>Granulocytes	
Braun 2002	Mice Lympho-sarcoma	Iscador sp., sc, 3/wk	up to 25	> 1000	14 d	No	*Peripheral blood:* Leukocytes, lymphocytes, monocytes, granulocytes<br>*Thymus*: Thymocytes	↑<br>↔	Leukocytes, lymphocytes, monocytes, thymocytes<br>Granulocytes	
Braun 2001	Mice Sarcoma	Helixor A, P, sc or ip, 3/wk	0.25 and 2.5	up to 8 and 36 resp.*	14 d	No	*Peripheral blood:* Leukocytes, lymphocytes, monocytes, granulocytes<br>*Thymus:* Size, thymocytes	↑	All parameters	
Braun 2001	Mice Lympho-sarcoma	Helixor A, P, sc or ip, 3/wk	0.25 and 2.5	up to 8 and 36 resp.*	14 d	No	*Peripheral blood:* Leukocytes, lymphocytes, monocytes, granulocytes<br>*Thymus:* Size, thymocytes	↑	All parameters	
Burger 2001[§§§]	Mice Various Ca.	Lektinol, ip or sc, 5/wk		0.3-300*	up to 4 wk	No	Safety: Body weight			Well tolerated
Elsässer-Beile 2001	Rats Carcino-genesis (chemical) in bladder	rML, intravesical, 2/wk		150 or 750	1 (3) mth	No	*Urinary bladder tissue*: Expression of INFγ, IL-10, Fas ligand (FasL)	↓<br>↔	IL-10 (slightly)<br>Other parameters	
Schaffrath 2001[§§§]	Mice Sarcoma, lympho-sarcoma	rML, sc, q2d		up to 150	14 d	No	*Peripheral blood:* Leukocytes, CD4, CD8 lymphocytes, NK-cells, activated monocytes/macrophages (Mac-3)<br>Safety: Daily: vitality, body weight	↑	Leukocytes, CD4, CD8 lymphocytes, NK cells, Mac-3	No obvious side effects
Mengs 2000	Mice Bladder Ca.	Lektinol, intravesical, 3/wk		120 and 1200*	4 wk	No	Safety: Daily, 2/wk clinical signs, body weight, ingestion; once bladder histology			Bladder: No lesions; local intolerance at 12000 ng/kg
Kubasova 1998	Mice Lung Ca.	ML I, sc or oral, 1/6 d		1-14000 (sc) 14000 - 100000 (oral)	19 d	RT (partly)	*Serum samples*: TNFα	↑	TNFα	

Author, reference	Animals, diagnosis	Preparation, application	Dosage per application		Treatment (follow-up) period	Conco-mitant therapy	Immune Parameter Investigated<br>*Compartment investigated*<br>Safety: Assessing frequency and method	Immune outcome compared to control group<br>↑ above, ↓ below,<br>↔ no difference	Safety outcome
			**Viscum album mg/kg**	**ML ng/kg**					
Weber 1998	Mice Melanoma	Lektinol iv, 4/wk		up to 150*	21 d	No	*Bronchoalveolar lavage*: Macrophages Mac-1<br>*Thymus:* CD4 CD8 thymocytes<br>Safety: Daily, 2/wk: mortality, clinical signs, body weight, ingestion; necropsy	↑ Mac-1, CD4 CD8 thymocytes	No local or systemic toxicity
Jurin 1993	Mice Sarcoma	Isorel M sc, daily	60		4 d	No	*Foreign skin graft* rejection (graft survival time)	↓ Treatment day 8-11<br>↔ Treatment day -4 – -1 or day 2-5	
Jurin 1993	Mice Sarcoma	Isorel M ip, once	140 or 1400		Day -1, 0 or 1 (4) d	No	*Immune response:* Influence on humoral response (PFC) to SRBC	↑ PFC (treatment day -1 or 0)<br>↔ PFC (treatment day 1)	
Beuth 1991	Mice Sarcoma, lympho-sarcoma	ML I sc, 2-4/wk		1 and 2	14 d	No	*Peritoneal macrophages:* Activity<br>*Thymus:* Size<br>*Spleen*: Size	↑ Macrophage activity, thymus, spleen (slightly)	
Kuttan 1990[§§§]	Mice Lymphoma, Ehrlich Ca.	Iscador M, im, q2d	83		up to 20 d	No	Safety: No details		No reduction of body weight, no bone marrow suppression
Raabe 1987	Mice Plasmo-cytoma	ML I, ip, once – 5 x		up to 50000[§§]	1 – 45 d	No	Safety: No details		Lethality at 10000 ng/kg in 20% of mice
Franz 1986	Mice Ehrlich Ca.	ML I, pre-incubation of tumour cells, ip, once		up to 1500 in 0.5 ml per mouse	1 (up to 30) d	No	Safety: No details		≥ 300 ng in 0.5 ml per mouse: increasing lethality; LD50: 33000 ng/kg
Berger 1983[§§§]	Rats, mice Various Ca.	Iscador M or P, sc, daily	median total: 10.5 - 750 mg		8 d – 6 wk	partly Cetraria	Safety: No details		No toxic effect
Seeger 1965[§§§]	Mice Ehrlich Ca.	Iscador, pre-incubation of tumour cells, sc, once	Up to 50 mg in 0.5 ml per mouse		1 (up to 186) d	No	Safety: No details		Toxic and lethal in higher dosages

Author, reference	Animals, diagnosis	Preparation, application	Dosage per application: Viscum album mg/kg	Dosage per application: ML ng/kg	Treatment (follow-up) period	Conco-mitant therapy	Immune Parameter Investigated *Compartment investigated* Safety: Assessing frequency and method	Immune outcome compared to control group ↑ above, ↓ below, ↔ no difference	Safety outcome
**Animals with sarcoid or sarcoma – therapeutic trials**									
Christen-Clottu 2010	Horses Equine Sarcoid	Iscador P, sc, 3/wk	up to 20 mg/horse		15 wk (12 mth)	No	Safety: Regular clinical examination, AE, local edema		Well tolerated, no ADRs, mild edema at inj. site
Blostin 2008	Cats Fibro-sarcoma	Iscador Qu, oral, 2/d	0.5 mg/cat		long-term (up to 5yr)	No	Safety: Tolerability§		Well tolerated, no ADRs§

§ Pre-post comparison; §§ partly pre-incubation of tumour cells with ML which reduced toxicity; §§§ experiments consist of several sub-experiments.

Abbreviations (see also additional file 2): d: day(s), DC: dendritic cell, DNA: desoxyribonucleic acid, DN: double negative, DP: double positive, gD2: herpes simplex virus glycoprotein 2, h: hour(s), ic: intracutaneous, ip: intraperitoneal, OVA: ovalbumin A, PBL: peripheral blood lymphocyte, PBM: peripheral blood monocyte, q2d: every other day, SCID: severe combined immunodeficiency

Reference List

Berger M, Schmähl D: **Studies on the tumor-inhibiting efficacy of Iscador in experimental animal tumors.** *J Cancer Res Clin Oncol* 1983, 262-265

Beuth J, Ko HL, Gabius H-J, Pulverer G: **Influence of treatment with the immunomodulatory effective dose of the β-galactoside-specific lectin from mistletoe on tumor colonization in BALB/c-mice for two experimental model systems.** *In Vivo* 1991, **5:**29-32

Beuth J, Ko HL, Schneider H, Tawadros S, Kasper HU, Zimst H, Schierholz JM: **Intratumoral application of standardized mistletoe extracts down regulates tumor weight via decreased cell proliferation, increased apoptosis and necrosis in a murine model.** *Anticancer Res* 2006, **26:**4451-4456

Beuth J, Ko HL, Tunggal L, Buss G, Jeljaszewicz J, Steuer MK, Pulverer G: **Immunaktive Wirkung von Mistellektin-1 in Abhängigkeit von der Dosierung.** *ArzneimForsch/DrugRes* 1994, **44(II):**1255-1258

Bloksma N, van Dijk H, Korst P, Willers J: **Cellular and humoral adjuvant activity of a mistletoe extract.** *Immunobiol* 1979, **156:**309-319

Blostin R, Faivre C: **Bénéfices du gui fermenté chez le chat après exérèse de fibrosarcome. Résultats d'une étude préliminaire.** *Phytotherapie* 2008, **6:**352-358

Braun JM, Ko HL, Schierholz JM, Beuth J: **Standardized mistletoe extract augments immune response and down-regulates local and metastatic tumor growth in murine models.** *Anticancer Res* 2002, **22:**4187-4190

Braun JM, Ko HL, Schierholz JM, Weir D, Blackwell CC, Beuth J: **Application of standardized mistletoe extracts augment immune response and down regulates metastatic organ colonization in murine models.** *Cancer Lett* 2001, **170:**25-31

Burger AM, Mengs U, Schuler JB, Fiebig HH: **Anticancer activity of an aqueous mistletoe extract (AME) in syngeneic murine tumor models.** *Anticancer Res* 2001, **21:**1965-1968

Christen-Clottu O, Klocke P, Burger D, Straub R, Gerber V: **Treatment of Clinically Diagnosed Equine Sarcoid with a Mistletoe Extract (Viscum album austriacus).** *J Vet Intern Med* 2010, **24:**1483-1489

Elsässer-Beile U, Ruhnau T, Freudenberg M, Wetterauer U, Mengs U: **Antitumoral effect of recombinant mistletoe lectin on chemically induced urinary bladder carcinogenesis in a rat model.** *Cancer* 2001, **91:**998-1004

Franz H: **Mistletoe lectins and their A and B chains.** *Oncology* 1986, **43:**23-34

Gren A: **Effects of Iscador preparations on the reactivity of mouse immune system.** *Neuroendocrinol Lett* 2009, **30:**153-157

Hajto T, Berki T, Boldizsar F, Nemeth P: **Galactoside-specific plant lectin, Viscum album agglutinin-I induces enhanced proliferation and apoptosis of murine thymocytes in vivo.** *Immunol Lett* 2003, **86:**23-27

Hajto T, Berki T, Palinkas L, Boldizsar F, Nemeth P: **Effects of mistletoe extract on murine thymocytes in vivo and on glucocorticoid-induced cell count reduction.** *Forsch Komplement Med (2006)* 2006, **13:**22-27

Hajto T, Berki T, Palinkas L, Boldizsar F, Nemeth P: **Investigation of the effect of mistletoe (Viscum album L.) extract Iscador on the proliferation and apoptosis of murine thymocytes.** *Arzneimittelforschung* 2006, **56:**441-446

Hajto T, Hostanska K, Weber K, Zinke H, Fischer J, Mengs U, Lentzen H, Saller R: **Effect of a recombinant lectin, *Viscum album* Agglutinin on the secretion of Interleukin-12 in cultured human peripheral blood mononuclear cells and on NK-cell-mediated cytotoxicity of rat splenocytes in vitro and in vivo.** *Nat Immun* 1998, **16:**34-46

Joshi SS, Gabius S, Gabius H-J: **Immunostimulatory/antitumor effects of mistletoe lectin [abstract].** *Proc Am Assoc Cancer Res* 1991, **32:**401

Joshi SS, Komanduri KC, Gabius S, Gabius H-J: **Immunotherapeutic effects of purified mistletoe lectin (ML-I) on murine large cell lymphoma.** In: *Lectins and Cancer*. Edited by Gabius H-J, Gabius S. Berlin, Heidelberg, New York, Springer-Verlag 1991, 207-216

Jurin M, Zarkovic N, Borovic S, Kissel D: **Immunomodulation by the *Viscum album* L. preparation Isorel and its antitumorous effects.** In: *Grundlagen der Misteltherapie. Aktueller Stand der Forschung und klinische Anwendung*. Edited by Scheer R, Becker H, Berg PA. Stuttgart, Hippokrates Verlag GmbH 1996, 315-324

Jurin M, Zarkovic N, Borovic S, Kissel D: ***Viscum album* L. preparation Isorel modifies the immune response in normal and in tumour-bearing mice.** *Anticancer Drugs* 1997, **8:**S27-S31

Jurin M, Zarkovic N, Hrzenjak M, Ilic Z: **Antitumorous and immunomodulatory effects of the *Viscum album* L. preparation Isorel.** *Oncology* 1993, **50:**393-398

Kubasova T, Pfüller U, Bojtor I, Köteles GJ: **Modulation of immune response by mistletoe lectin I as detected on tumour model *in vivo*.** In: *COST 98. Effects of antinutrients on the nutritional value of legume diets*. Edited by Bardocz S, Pfüller U, Pusztai A. Luxembourg, Office for Official Publications of the European Communities 1998, 202-207

Kuttan G, Kuttan R: **Immunological mechanism of action of the tumor reducing peptide from mistletoe extract (NSC 635089) cellular proliferation.** *Cancer Lett* 1992, 123-130

Kuttan G, Kuttan R: **Immunomodulatory activity of a peptide isolated from *Viscum album* extract (NSC 635089).** *Immunol Invest* 1992, **21:**285-296

Kuttan G, Kuttan R: **Reduction of leukopenia in mice by "*Viscum album*" administration during radiation and chemotherapy.** *Tumori* 1993, 74-76

Kuttan G, Kuttan V, Kuttan R: **Effect of a preparation from *Viscum album* on tumor development in vitro and in mice.** *Journal of Ethnopharmacology* 1990, **29:**35-41

Lavelle EC, Grant G, Pfüller G, O'Hagan DT: **Immunological implications of the use of plant lectins for drug and vaccine targeting to the gastrointestinal tract.** *J Drug Target* 2004, **12:**89-95

Lavelle EC, Grant G, Pusztai A, Pfüller U, Leavy O, McNeela E, Mills KH, O'Hagan DT: **Mistletoe lectins enhance immune responses to intranasally co-administered herpes simplex virus glycoprotein D2.** *Immunology* 2002, **107:**268-274

Lavelle EC, Grant G, Pusztai A, Pfüller U, O'Hagan DT: **The identification of plant lectins with mucosal adjuvant activity.** *Immunology* 2001, **102:**77-86

Mengs U, Schwarz T, Bulitta M, Weber K: **Antitumoral effects of an intravesically applied aqueous mistletoe extract on urinary bladder carcinoma MB49 in mice.** *Anticancer Res* 2000, **20:**3565-3568

Raabe F, Storch H: **Untersuchungen zur Therapie des Maus-Plasmozytoms mit Mistellektin I.** *Wiss Z Karl-Marx-Univ Leipz Math-Naturwiss R* 1987, **36:**535-543

Rentea R, Lyon E, Hunter R: **Biologic properties of Iscador: A *Viscum album* preperation. I. Hyperplasia of the thymic cortex and accelerated regeneration of hematopoietic cells following x-irradiation.** *Lab Invest* 1981, **44:**43-48

Rostock M, Huber R, Greiner T, Fritz P, Scheer R, Schueler J, Fiebig HH: **Anticancer activity of a lectin-rich mistletoe extract injected intratumorally into human pancreatic cancer xenografts.** *Anticancer Res* 2005, **25:**1969-1975

Schaffrath B, Mengs U, Schwarz T, Hilgers RD, Beuth J, Möckel B, Lentzen H, Gerstmayer B: **Anticancer activity of rViscumin (recombinant mistletoe lectin) in tumor colonization models with immunocompetent mice.** *Anticancer Res* 2001, **21:**3981-3987

Seeger PG: **Über die Wirkung von Mistelextrakten (Iscador und Plenosol).** *Erfahrungsheilkunde* 1965, **14:**149-174

Seifert G, Jesse P, Längler A, Reindl T, Lüth M, Lobitz S, Henze G, Prokop A, Lode HN: **Molecular mechanisms of mistletoe plant extract-induced apoptosis in acute lymphoblastic leukemia in vivo and in vitro.** *Cancer Lett* 2008, **264:**218-228

Stettler B, Probst F, Felder K: **Effects of repeated intraperitoneal injections of *Viscum album*-extracts on mouse thymus and lymph nodes. A contribution to the problem of antigenic influences on the thymus.** *PhD Thesis.* Medizinische Fakultät der Universität Bern; 1978.

Thies A, Dautel P, Meyer A, Pfuller U, Schumacher U: **Low-dose mistletoe lectin-I reduces melanoma growth and spread in a scid mouse xenograft model.** *Br J Cancer* 2008, **98:**106-112

Van Huyen JP, Delignat S, Bayry J, Kazatchkine MD, Bruneval P, Nicoletti A, Kaveri SV: **Interleukin-12 is associated with the in vivo anti-tumor effect of mistletoe extracts in B16 mouse melanoma.** *Cancer Lett* 2006, **243:**32-37

Weber K, Mengs U, Schwarz T, Hajto T, Hostanska K, Allen TR, Weyhenmeyer R, Lentzen H: **Effects of a standardized mistletoe preparation on metastatic B16 melanoma colonization in murine lungs.** *ArzneimForsch/DrugRes* 1998, **48:**497-502
